# Investments in Childhood Community Resources and Subsequent Adult Health Outcomes

**DOI:** 10.1001/jamanetworkopen.2026.16711

**Published:** 2026-06-04

**Authors:** Jaime La Charite, Rebecca Dudovitz, Kristen Choi, Keren Chen, Nicholas J. Jackson, Teryl Nuckols, Narayan Sastry, Adam Schickedanz, Mitchell D. Wong, Frederick J. Zimmerman

**Affiliations:** 1 Division of General Internal Medicine and Health Services Research, David Geffen School of Medicine at UCLA, University of California, Los Angeles; 2 Department of Pediatrics, University of California, Los Angeles; 3 School of Nursing, University of California, Los Angeles; 4 Division of General Internal Medicine and Health Services Research, Department of Medicine Statistics Core (DOMStat), David Geffen School of Medicine at UCLA, University of California, Los Angeles; 5 Division of General Internal Medicine, Cedars-Sinai Medical Center, Los Angeles, California; 6 Institute for Social Research, University of Michigan, Ann Arbor; 7 Department of Pediatrics, University of California, Los Angeles; 8 Department of Health Policy and Management, Fielding School of Public Health, University of California, Los Angeles

## Abstract

**Importance:**

Community resources may benefit children’s long-term health, but the lasting impact of public spending in childhood is unclear. Identifying policies to address residential wealth and opportunity disparities could promote long-term health equity.

**Objective:**

To assess whether childhood exposure to public spending on community resources (public primary and secondary education, libraries, parks and recreation, and community development and housing) is associated with subsequent adult health.

**Design, Setting, and Participants:**

This retrospective cohort study used linked national datasets from 1977 through 2019. US cities with at least 150 000 residents in 1980 were included. Childhood public finance data covered 1977 through 2017, and adult health was measured in 2019. Included adults were in the 2019 Panel Study of Income Dynamics, were born between 1960 and 2000, and lived in a medium to large city before 18 years of age. Analyses were performed from August 2024 to March 2026.

**Exposure:**

Total per capita operational spending on community resources, summed across city, county, and school district levels for the year and city when the respondent was 9 years of age.

**Main Outcomes and Measures:**

The main outcome was overall adult health rated as fair or poor. Secondary outcomes included cardiovascular disease (CVD), anxiety, and depression diagnoses. Data were analyzed using weighted linear probability models adjusted for demographics. Estimated margins compared the 25th and 75th percentiles of spending.

**Results:**

Among 2214 adults (mean [SD] age in 2019, 38.9 [10.2] years; 1223 [52%] female), 389 (17%) reported fair or poor health, 458 (22%) reported CVD, and 184 (10%) reported anxiety or depression. A 1% increase in childhood community resource spending was associated with a 0.20 (95% CI, 0.04-0.35) percentage point decrease in adult fair or poor health and a 0.25 (95% CI, 0.07-0.44) percentage point decrease in adult CVD. To contextualize the magnitude, shifting from the 25th to 75th spending percentile decreased the estimated probability of reporting adult fair or poor health from 19.38% to 12.87%, a reduction of 6.51 (95% CI, 1.38-11.64) percentage points. A 1% increase in education (0.15 [95% CI, 0.02-0.29] percentage points) or library (0.05 [95% CI, 0.01-0.10] percentage points) spending was correlated with better overall health. Education (0.23 [95% CI, 0.07-0.39] percentage points) and community development and housing (0.04 [95% CI, 0.01-0.08] percentage points) spending were associated with lower CVD. No association was found for overall community resource spending or its individual four component sectors and anxiety or depression.

**Conclusions and Relevance:**

In this cohort study of US urban adults, greater childhood exposure to community resource spending was associated with improved overall and cardiovascular health in adulthood. Variation in public spending levels may partially explain geographic differences in US health outcomes.

## Key Points

**Question:**

Is there an association between childhood exposure to public spending on community resources (public primary and secondary education, libraries, parks and recreation, and community development and housing) and subsequent adult health?

**Findings:**

This cohort study of 2214 adults found that greater exposure to childhood community resource spending was associated with fewer reports of fair or poor overall health and cardiovascular disease diagnoses as an adult. No association was found with anxiety or depression diagnoses.

**Meaning:**

These findings suggest that policymakers and government officials should consider the possible long-term beneficial health impacts of community resource investments.

## Teaser

This cohort study investigates whether childhood exposure to public spending on community resources is associated with subsequent adult health.

## Introduction

Environments where children live, play, and attend school can influence their development, health, and well-being.^1^ Better neighborhood conditions and resources are associated with improved child physical health, increased life expectancy, and possible protection against the negative health consequences of poverty.^2,3,4,5,6,7^ However, disparities in neighborhood conditions and resources that support child development can also sustain inequalities related to race, ethnicity, and income.^8,9,10,11^ If neighborhoods influence health, policymakers must identify actions that mitigate or reverse the impact of differential wealth and opportunity distributions across neighborhoods.^1,7,11^

Local public spending at the city, county, and school district levels on community resources is a potential lever for improving childhood environments, development, and lifelong health. Public investment varies between low- and high-income areas.^12^ For example, public education, often funded by property taxes, can benefit wealthier neighborhoods that generate more revenue compared with less wealthy districts, which tend to represent more disadvantaged students and students of color.^13^ Prior research suggests greater public spending on child- and family-relevant resources—including public education, libraries, parks and recreation, and community development and housing (family-focused community resources [FFCRs])—is associated with improved area-level health metrics, including county health rankings, measures of well-being, prevalence of chronic conditions, and mortality rates.^14,15,16,17,18^ These studies primarily measure contemporaneous adult health outcomes cross-sectionally at the population level. However, we are not aware of a study using individual-level data that has examined whether childhood exposure to FFCR spending has health benefits that persist into adulthood. Investing in community resources that benefit children may shape long-term positive health trajectories.

This study evaluates the longitudinal association between childhood exposure to local public spending on FFCRs and adult health outcomes in US cities; 86% of the US population resides in urban or suburban cities.^19^ We examined how childhood FFCR spending from 1977 through 2017 was associated with overall health in young to middle adulthood, as reported in 2019. Secondary outcomes include lifetime diagnoses of cardiovascular disease (CVD), anxiety, and depression. We hypothesized that greater childhood exposure to FFCR spending is associated with improved adult health. Secondary analyses evaluated associations between individual FFCR categories—public education, libraries, parks and recreation, and community development and housing—with the adult health outcomes.

## Methods

### Study Design

We conducted a retrospective cohort study using linked national datasets spanning from 1977 through 2019. Method details not given here are reported in the eMethods in Supplement 1. The University of California, Los Angeles, institutional review board approved this study and waived the requirement for obtaining informed consent because the research presents no more than minimal risk of harm, and the secondary data we analyzed does not contain identifiers that would allow for further consent. We followed the Strengthening the Reporting of Observational Studies in Epidemiology (STROBE) reporting guideline.^20^

### Setting

The study included US cities with at least 150 000 residents in 1980, where roughly 46 million US children (72%) lived.^21^ Childhood public finance data spanned from 1977 through 2017. Adult health outcomes were measured in 2019.

### Data Sources and Dataset Construction

#### Fiscally Standardized Cities Database (Public Spending Independent Variable and Covariate)

The Fiscally Standardized Cities database integrates municipal, county, special district, and school district finance data from the US Census Bureau Census of Government Finance and Annual Surveys since 1977.^1^ To calculate standardized local government fiscal estimates, city expenditures were combined with proportions from counties, school districts, and special districts allocated for the total residents or students in the central city.^1^ The dataset included 212 cities selected based on criteria defined by the dataset administrators, including population size, the extent of population decline, and poverty rates.^1^

#### Panel Study of Income Dynamics (Health Dependent Variables and Individual-Level Covariates)

The Panel Study of Income Dynamics (PSID)^22^ is a nationally representative panel survey of individuals and families that began in 1968.^23^ The PSID conducted interviews with panel members every 1 to 2 years. We drew health outcome data from the PSID 2019 Core Survey (response rate 9569 of 10 837 [88%]).^23^We extracted childhood and adult demographic covariate data from waves of PSID Core Surveys from 1977 through 2019. We used the PSID unique identification number to link a respondent’s longitudinal PSID data across their 1977 through 2019 PSID Core Surveys.

#### Data Source Linkage

We used the Integrated Public Use Microdata Series National Historical Geographic Information System census data hub to construct city-level demographic covariates.^24,25^ We used Federal Information Processing Standard place codes corresponding to the PSID respondents’ childhood city at 9 years of age (or the closest age to 9 years if data were unavailable) to link the data sources.

### Participants

**Figure 1.**
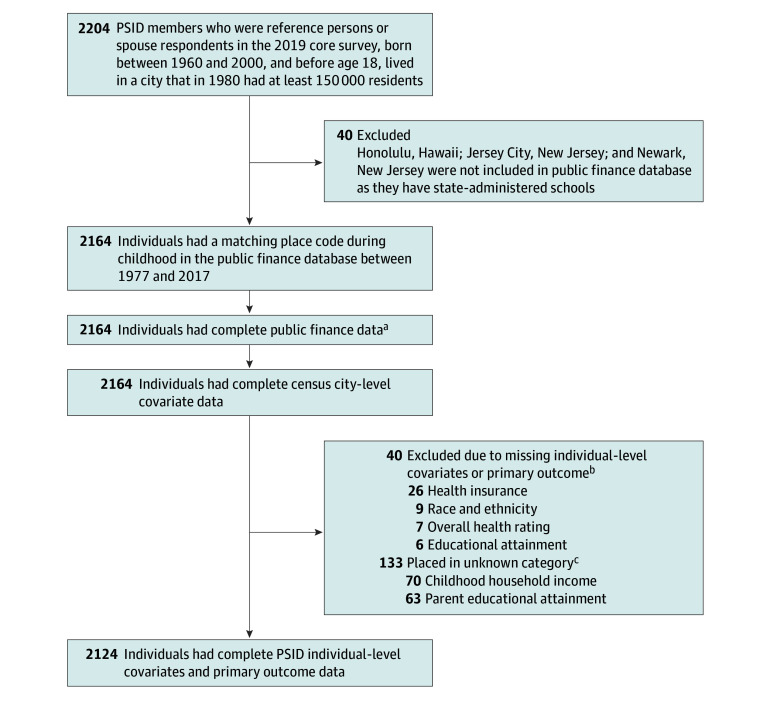
Sample Flow Diagram for Constructing the Analytic Sample From the 2019 PSID Core Survey PSID represents Panel Study of Income Dynamics. ^a^Fiscally Standardized Cities database administrators imputed missing public spending variables. ^b^Eight individuals were missing more than 1 variable. ^c^Missing values placed in the unknown category.

We set eligibility criteria for study participants as follows. Participants (1) lived in a household within the PSID 2019 national probability sample, (2) were the adult reference person (household member with primary financial responsibility, whether they lived alone or with others) or the spouse or partner in the 2019 PSID Core Survey, (3) were a PSID sample member since childhood, (4) were born between 1960 and 2000, and (5) resided, before age 18 years, for at least 1 year between 1977 and 2017 in 1 of 95 cities with a population of at least 150 000 in 1980 (Figure 1).

### Variables

#### Exposure Variable: Public Spending (Childhood)

We summed the standardized local government per capita operational spending estimates on FFCRs for the linkage year (city and year when respondent was 9 years of age). FFCR categories included primary and secondary education, libraries, parks and recreation, and community development and housing (eTable 1 in Supplement 1). These categories are relevant to families and children while excluding health care spending. The age of 9 years marks middle childhood, when children are of school age, and since spending is relatively stable over 10-year periods, it can approximate average spending levels over the course of childhood from birth to age 18 years.^26^ Spending estimates were inflated to 2019 dollars and log-transformed to address skewness and to reflect diminishing returns.

#### Adult Health Outcome Variables (Young to Middle Adulthood)

The primary outcome was overall health, self-reported or by spouse or partner proxy, rated on a 5-point scale and dichotomized (fair or poor vs good, very good, or excellent), which is a well validated estimator of morbidity and mortality.^5,27^ Secondary outcomes included self- or proxy-reported lifetime CVD diagnosis (hypertension, stroke, heart attack, or coronary artery disease) or mental health condition (depression or anxiety).

#### Covariates

We adjusted for childhood city-level characteristics, including population size and density, median gross rent (2019 dollars), and the percentage of residents under 18 years of age, who had a high school diploma, lived below the federal poverty level (FPL), were unemployed, were non-Hispanic White, or were foreign born. Race and ethnicity were assessed in this study to adjust for ways racism can influence childhood environments and health.^28^ Categories included Asian or Pacific Islander, Black, Hispanic or Latinx, non-Hispanic White, and multiracial or other. The other category included non-Hispanic American Indian, non-Hispanic Alaskan Native, non-Hispanic Multiracial, or, if the respondent selected other as their race. We also controlled for operational spending on remaining non-FFCR categories, such as hospitals (eTable 1 in Supplement 1). We adjusted for individual-level childhood household income relative to FPL, highest parental educational attainment, and a childhood residential move. We controlled for individual-level (adulthood) age in 2019, sex, race, ethnicity, marital status, educational attainment, household income, and health insurance coverage and whether the information was self- or proxy-reported by a household family member.

### Statistical Analysis

For descriptive analyses, we calculated frequencies and survey-weighted percentages for the categorical variables and weighted mean and SD for the continuous variables. For multivariable primary analyses, we used a linear probability model to regress dichotomized adult overall health on the natural log of per capita operational spending on FFCRs during childhood (in 2019 dollars), adjusting for covariates. We applied longitudinal survey weights to account for unequal selection probabilities, complex sampling design, and differential attrition. We used 2-sided tests, and *P* < .05 was considered statistically significant.

#### Multivariable Secondary Analyses

We used separate linear probability models to regress CVD and anxiety or depression on the natural log of per capita operational FFCR spending. Separate linear probability models regressed the 3 health outcomes on the natural log of per capita operational spending for each of the 4 FFCR subcategories loaded into the same model.

#### Postestimation and Missing Variables

We used margins to estimate probabilities of health outcomes at the 25th and 75th percentiles of logged FFCR spending. We created unknown categories for missing childhood variables and used complete case analysis due to low missing data (2%).

#### Sensitivity Analyses

We tested alternative specifications of outcomes, exposures, and covariates and used logistic regression. We tested for moderation by age, childhood residential moves, presence of a county overlay, and district structure.

Statistical analyses were conducted in Stata SE, version 17.0 (StataCorp LLC). Data were acquired and cleaned from November 2022 to July 2024 and analyzed from August 2024 to March 2026.

## Results

### Sample Characteristics

The final sample consisted of 2124 adults (aged 19-59 years) who lived in the study cities during their childhood (Table 1; eTable 2 in Supplement 1). Their mean (SD) age was 38.9 (10.2) years in 2019, 1223 (52%) were female, 901 (48%) were male, and 1726 (87%) had household incomes above 100% of the FPL. The racial and ethnic groups included 21 (2%) Asian or Pacific Islander, 1335 (33%) Black, 177 (14%) Hispanic or Latinx, and 483 (47%) non-Hispanic White individuals.

**Table 1.** Characteristics of Eligible Respondents in the Weighted Study Sample

Characteristic	Respondents, No. (weighted %), n = 2124
Childhood individual-level variable	
Parent highest educational attainment	
Outside the country	67 (6)
<High school	215 (7)
High school graduate or GED	706 (27)
Some college or vocational	529 (23)
College graduate	359 (21)
Graduate school	191 (14)
Unknown	57 (2)
Childhood household income, % FPL	
<100	654 (20)
100-199	524 (22)
200-299	360 (18)
300-399	239 (17)
≥400	280 (22)
Unknown	67 (2)
Move during childhood	
No	1708 (79)
Yes	416 (21)
Adulthood individual-level variables	
Age in 2019, mean (SD) [range], y	38.9 (10.2) [19-59]
Sex	
Female	1223 (52)
Male	901 (48)
Race and ethnicity	
Asian or Pacific Islander	21 (2)
Black	1335 (33)
Hispanic or Latinx	177 (14)
Non-Hispanic White	483 (47)
Other or multiracial^a^	108 (4)
Educational attainment	
<High school	221 (7)
High school graduate or GED	674 (28)
Some college or vocational	683 (30)
College graduate	294 (18)
Graduate school	252 (17)
Marital status	
Married	677 (43)
Never married	1096 (42)
Widowed, divorced, annulled, or separated	351 (14)
Adulthood household income, % FPL	
<100	398 (13)
100-199	436 (17)
200-299	339 (14)
300-399	292 (14)
≥400%	659 (43)
Family health insurance	
Yes	1863 (91)
Respondent: self or by proxy	
Self	1976 (89)
Overall health rating	
Fair or poor health	389 (17)
Cardiovascular disease diagnosis	
Yes	458 (22)
Anxiety or depression diagnosis	
Yes	184 (10)
City demographics	
City population size	
Midsize (population 100 000-250 000)	470 (24)
Large (population >250 000)	1654 (76)
Percentage of city population, mean (SD)	
<18 y	26 (3)
≥25 y Of age with high school diploma or GED	48 (21)
Below the FPL	17 (5)
Unemployed	8 (3)
Non-Hispanic White	56 (20)
Foreign born	12 (12)
Population density, mean (SD)^b^	0.02 (0.04)
Median gross rent, mean (SD), $	897 (194)
City spending variables, mean (SD)^c^	
Total, $	4063 (1345)
FFCR, $	1725 (428)
Proportion of total on FFCRs^d^	0.44 (0.07)
Education, $	1424 (364)
Library, $	37 (17)
Parks and recreation, $	114 (60)
Community development and housing, $	150 (116)

Abbreviations: GED, General Educational Development; FFCRs, family-focused community resources; FPL, federal poverty level.

^a^ Other race category includes respondent selecting Alaska Native, American Indian, multiracial, or other.

^b^ Population density is the mean population per square meter.

^c^ All dollar amounts are per capita and adjusted to be comparable to 2019 dollars.

^d^ FFCRs include public primary and secondary education, libraries, parks and recreation, and community development and housing.

#### Childhood

Childhood data linkage occurred at 9 years of for 45% of the sample (n = 966), at ages 8 or 10 for 17% (n = 354), between ages 1 and 7 for 14% (n = 299), and at ages 11 to 17 for 24% (n = 505). Most respondents (1470 [80%]) reported childhood household incomes at or above 100% of the FPL, and 416 respondents (21%) reported moving before 17 years of age.

#### Childhood City

For the sample cities, the mean (SD) percentage of the population living below the FPL was 17% (5%) and who identified as a race other than non-Hispanic White was 44% (20%). The mean (SD) total per capita operational spending budget for the fiscally standardized cities was $4063 ($1345), with 44% allocated to FFCRs (mean [SD], $1725 [$428]); most (83%) of the FFCR spending (mean [SD], $1424 [$364]) was allocated to public education.

#### Adulthood

For the health outcomes, 389 of respondents (17%) reported fair or poor health. In addition, 458 respondents (22%) reported CVD (427 cases [93%] were hypertension), and 184 respondents (10%) reported anxiety or depression.

### Primary Analysis

**Figure 2.**
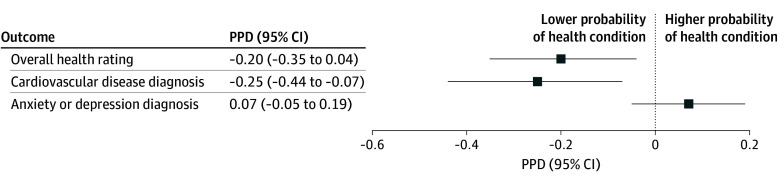
Forest Plot Illustrating Percentage Point Differences (PPDs) in Adult Health Outcomes Associated With Family-Focused Community Resources (FFCRs) Spending PPD per 1% increase in public spending on FFCRs. The independent variable was operational spending per capita on FFRCs (public primary and secondary education, libraries, parks and recreation, community development, and housing) on a natural log scale. Regression models were weighted using the Panel Study of Income Dynamics 2019 longitudinal survey weight. Models were adjusted for variables given in Table 1.

After covariate adjustment and survey weights, exposure to a relative 1% increase in childhood FFCR spending above baseline was associated with a 0.20 (95% CI, 0.04-0.35) percentage point decrease in the probability of reporting fair or poor adult health (Figure 2, Table 2; eTable 3 in Supplement 1 for unweighted and unadjusted models). Comparing the 25th to the 75th spending percentile in FFCR ($584 per capita difference), the estimated probability of reporting adult fair or poor health decreased from 19.38% to 12.87%, a reduction of 6.51 (95% CI, 1.38-11.64) percentage points.

**Table 2.** Adult Health Outcomes and Fiscally Standardized City Operational Expenditures per Capita for FFCRs

	Health outcome^a^					
	Fair or poor health (n = 2124)		Cardiovascular disease diagnosis (n = 2124)		Anxiety or depression diagnosis (n = 2120)	
	PPD (95% CI)	*P* value	PPD (95% CI)	*P* value	PPD (95% CI)	*P* value
Public spending variables						
FFCR spending, ln scale^b^	−0.20 (−0.35 to −0.04)	.01	−0.25 (−0.44 to −0.07)	.007	0.07 (−0.05 to 0.19)	.27
Spending on other categories, by quartile^c^	[Reference]	NA	[Reference]	NA	[Reference]	NA
2nd	−0.01 (−0.09 to 0.06)	.72	0.05 (−0.04 to 0.13)	.26	−0.06 (−0.12 to 0.00)	.06
3rd	0.09 (0.00 to 0.17)	.04	0.03 (−0.06 to 0.12)	.48	−0.05 (−0.11 to 0.02)	.17
4th	0.08 (−0.02 to 0.18)	.13	0.07 (−0.04 to 0.18)	.19	−0.07 (−0.15 to 0.01)	.06
Childhood individual-level demographic variables						
Parent highest educational attainment						
Outside the country	0.09 (−0.06 to 0.23)	.23	0.06 (−0.06 to 0.18)	.34	0.01 (−0.07 to 0.09)	.76
<High school	0.11 (−0.00 to 0.23)	.05	0.07 (−0.04 to 0.19)	.20	−0.02 (−0.08 to 0.04)	.55
High school graduate	[Reference]	NA	[Reference]	NA	[Reference]	NA
Some college or vocational	−0.04 (−0.10 to 0.03)	.31	0.02 (−0.06 to 0.11)	.58	−0.00 (−0.06 to 0.05)	.93
College graduate	0.01 (−0.07 to 0.08)	.90	−0.06 (0.14 to 0.02)	.16	−0.01 (−0.07 to0.05)	.69
Graduate school	−0.00 (−0.09 to 0.09)	.94	−0.06 (−0.15 to 0.04)	.23	−0.01 (−0.08 to0.07)	.89
Unknown	−0.06 (−0.24 to 0.12)	.51	0.01 (−0.19 to 0.22)	.91	0.01 (−0.07 to 0.08)	.88
Childhood household income, % FPL						
<100	[Reference]	NA	[Reference]	NA	[Reference]	NA
100-199	−0.05 (−0.12 to 0.03)	.26	−0.04 (−0.12 to 0.05)	.41	0.02 (−0.02 to 0.07)	.36
200-299	−0.04 (−0.13 to 0.04)	.30	0.01 (−0.08 to 0.09)	.89	−0.02 (−0.07 to 0.03)	.45
300-399	−0.03 (−0.12 to 0.05)	.46	−0.03 (−0.13 to0.06)	.49	0.05 (−0.02 to 0.11)	.15
≥400%	−0.02 (−0.11 to 0.08)	.71	0.02 (−0.08 to 0.11)	.76	0.09 (0.02 to0.15)	.01
Unknown	−0.02 (−0.18 to 0.14)	.80	−0.16 (−0.27 to −0.04)	.01	−0.01 (−0.09 to 0.06)	.79
Moved during childhood						
No	[Reference]	NA	[Reference]	NA	[Reference]	NA
Yes	0.05 (−0.01 to 0.11)	.13	0.09 (0.01 to 0.16)	.02	0.03 (−0.01 to 0.07)	.19
Childhood city-level demographic variables						
City population size						
Large (population >250 000)	[Reference]	NA	[Reference]	NA	[Reference]	NA
Midsize (population 100 000-250 000)	0.01 (−0.06 to 0.08)	.75	0.02 (−0.05 to 0.10)	.57	0.04 (−0.02 to 0.09)	.19
City demographics						
Percentage of population <18 y of age	0.00 (−0.04 to 0.05)	.93	0.00 (−0.04 to 0.05)	.92	0.01 (−0.03 to 0.05)	.67
Percentage of population ≥25 y of age and at least high school diploma or GED, below median	[Reference]	NA	[Reference]	NA	[Reference]	NA
Above median	0.03 (−0.03 to 0.10)	.32	−0.03 (−0.10 to 0.04)	.41	−0.01 (−0.06 to 0.04)	.63
Percentage of population below FPL	−0.03 (−0.08 to 0.01)	.18	−0.03 (−0.08 to 0.02)	.28	0.04 (0.00 to 0.08)	.03
Percentage of population that is unemployed	−0.02 (−0.09 to 0.04)	.45	0.05 (−0.03 to 0.13)	.24	−0.05 (−0.10 to −0.00)	.04
Percentage of population that is non-Hispanic white	−0.01 (−0.02 to 0.00)	.15	−0.00 (−0.01 to 0.01)	.82	0.00 (−0.01 to 0.01)	.52
Percentage of population that is foreign born, ln scale	−0.00 (−0.04 to 0.04)	.87	−0.01 (−0.05 to 0.03)	.59	−0.02 (−0.05 to 0.01)	.19
Population density, ln scale	−0.01 (−0.03 to 0.02)	.58	0.01 (−0.02 to 0.03)	.68	−0.00 (−0.02 to 0.02)	.90
Median rent, adjusted to 2019, ln scale	0.02 (−0.14 to 0.18)	.79	−0.10 (−0.28 to 0.08)	.27	0.17 (0.01 to 0.32)	.04
Adult individual-level variables						
Age in 2019, y	0.00 (0.00 to 0.01)	.05	0.01 (0.00 to 0.01)	<.001	0.00 (−0.003 to 0.004)	.80
Sex						
Female	−0.02 (−0.07 to 0.03)	.41	−0.05 (−0.11 to −0.00)	.04	0.07 (0.04 to 0.11)	<.001
Male	[Reference]	NA	[Reference]	NA	[Reference]	NA
Race and ethnicity						
Asian or Pacific Islander	0.04 (−0.12 to 0.20)	.62	0.12 (−0.06 to 0.30)	.19	0.03 (−0.05 to 0.12)	.48
Black	[Reference]	NA	[Reference]	NA	[Reference]	NA
Hispanic or Latinx	−0.04 (−0.13 to 0.06)	.46	−0.02 (−0.12 to 0.08)	.65	0.03 (−0.03 to 0.08)	.41
White	−0.03 (−0.10 to 0.05)	.48	−0.02 (−0.09 to 0.06)	.66	0.12 (0.07 to 0.17)	<.001
Other or multiracial	−0.07 (−0.16 to 0.03)	.19	−0.04 (−0.15 to 0.07)	.46	0.04 (−0.04 to 0.12)	.34
Educational attainment						
<High school	0.05 (−0.06 to 0.16)	.33	0.03 (−0.08 to 0.14)	.56	−0.02 (−0.08 to 0.04)	.48
High school graduate	[Reference]	NA	[Reference]	NA	[Reference]	NA
Some college or vocational	0.02 (−0.05 to 0.09)	.57	0.03 (−0.05 to 0.10)	.46	0.01 (−0.04 to 0.06)	.60
College graduate	−0.00 (−0.08 to 0.08)	.95	−0.01 (−0.10 to 0.07)	.76	−0.01 (−0.07 to 0.05)	.79
Graduate school	−0.06 (−0.13 to 0.02)	.13	−0.01 (−0.10 to 0.09)	.91	0.02 (−0.05 to 0.09)	.59
Marital status						
Never married	[Reference]	NA	[Reference]	NA	[Reference]	NA
Married	0.01 (−0.05 to 0.07)	.70	−0.00 (−0.07 to 0.06)	.92	−0.02 (−0.06 to 0.03)	.45
Widowed, divorced, annulled, or separated	0.03 (−0.04 to 0.11)	.38	−0.01 (−0.09 to 0.08)	.87	0.05 (−0.01 to 0.11)	.10
Ratio of household income to poverty limit, log scale	−0.18 (−0.24 to −0.12)	<.001	−0.13 (−0.20 to −0.06)	<.001	−0.07 (−0.12 to −0.02)	.007
Has family health insurance	0.03 (−0.06 to 0.12)	.46	0.13 (0.05 to 0.22)	.002	0.03 (−0.01 to 0.08)	.16
Self-response	0.01 (−0.07 to 0.09)	.76	0.04 (−0.06 to 0.14)	.45	−0.01 (−0.07 to 0.06)	.78

Abbreviations: GED, General Educational Development; FFCR, family-focused community resource; FPL, federal poverty limit; ln, natural log; NA, not applicable; PPD, percentage point difference.

^a^ Linear probability regression models were weighted using the Panel Study of Income Dynamics 2019 longitudinal survey weight; values are PPDs in the outcome for a 1% increase in FFCR spending.

^b^ Included operational spending per capita on public primary and secondary education, libraries, parks and recreation, and community development and housing.

^c^ Non–community-based support resource sectors included the residual operational spending on other welfare, higher education, hospitals, public health, transportation, public safety, natural resources, sewage, solid waste management, governmental administration, interest on general debt, utilities, liquor store, and employee retirement trust expenditures after removing the operational spending on community-based support resources.

### Secondary Outcomes

Exposure to a 1% increase in childhood FFCR spending was associated with a 0.25 (95% CI, 0.07-0.44) percentage point decrease in the probability of reporting adult CVD (Figure 2, Table 2; eTable 3 in Supplement 1 for unweighted and unadjusted models). Comparing the 25th to the 75th percentiles in FFCR spending, the estimated probability of reporting adult CVD decreased from 24.85% to 16.40%, a reduction of 8.45 (95% CI, 2.35-14.55) percentage points. No association was found between FFCR spending and lifetime anxiety or depression diagnoses (0.07 [95% CI, −0.05 to 0.19] percentage points).

### Sector-Specific Spending

When we stratified FFCR spending by the 4 sectors, associations remained for public primary and secondary education, libraries, and community development and housing. Childhood exposure to a 1% increase in public primary and secondary education spending was associated with 0.15 (95% CI, 0.02-0.29) and 0.23 (95% CI, 0.07-0.39) percentage point decreases in reporting fair or poor adult health and CVD, respectively (Table 3). Comparing the 25th to the 75th percentiles of education spending ($456 per capita difference), the estimated probability of reporting adult fair or poor health decreased from 19.03% to 13.62%, a reduction of 5.42 (95% CI, 1.06-9.78) percentage points, and CVD decreased from 24.21% to 17.68%, a reduction of 6.52 (95% CI, 1.40-11.64) percentage points.

**Table 3.** Adult Health Outcomes and Fiscally Standardized City Operational Expenditures per Capita for FFCRs by Sector

Public spending variable^a^	Health outcome^b^					
	Fair or poor health (n = 2124)		Cardiovascular disease diagnosis (n = 2124)		Anxiety or depression diagnosis (n = 2120)	
	PPD (95% CI)^c^	*P* value	PPD (95% CI)^c^	*P* value	PPD (95% CI)^c^	*P* value
Public education	−0.15 (−0.29 to −0.02)	.03	−0.23 (−0.39 to −0.07)	.01	0.07 (−0.04 to 0.19)	.22
Libraries	−0.05 (−0.10 to −0.01)	.03	0.00 (−0.04 to 0.05)	.94	0.00 (−0.03 to 0.03)	.97
Parks and recreation	−0.05 (−0.10 to 0.01)	.10	−0.01 (−0.07 to 0.05)	.80	0.01 (−0.04 to 0.05)	.80
Community development and housing	0.01 (−0.01 to 0.04)	.22	−0.04 (−0.08 to −0.01)	.01	0.01 (−0.02 to2 0.03)	.65

Abbreviations: FFCR, family-focused community resource; PPD, percentage point difference.

^a^ The spending exposure variable was spending per capita on a natural log scale.

^b^ Linear probability regression models were weighted using the Panel Study of Income Dynamics 2019 longitudinal survey weight; values are PPDs in the outcome for a 1% increase in FFCR spending.

^c^ Analyses adjusted for covariates given in Table 2. All spending categories were included simultaneously in the model; therefore, PPDs reflect the association independent of the other 3 spending sectors.

Childhood exposure to a 1% increase in library spending was associated with a 0.05 (95% CI, 0.01-0.10) percentage point decrease in adult fair or poor health. Comparing the 25th to the 75th percentile in library spending ($19 per capita difference), the estimated probability of reporting adult fair or poor health decreased from 18.13% to 14.71%, a reduction of 3.41 (95% CI, 0.98-5.85) percentage points. Childhood exposure to a 1% increase in community development and housing spending was associated with a 0.04 (95% CI, 0.01-0.08) percentage point decrease in the probability of reporting CVD. Comparing the 25th to the 75th percentile of community development and housing spending ($175 per capita difference), the estimated probability of reporting adult CVD decreased from 23.21% to17.93%, a reduction of 5.28 (95% CI, 1.25-9.31) percentage points.

Childhood parks and recreation spending showed no associations (eg, for fair or poor health, −0.05 [95% CI, −0.10 to 0.01] percentage points). No spending category was associated with anxiety or depression diagnoses (eg, for public education, 0.07 [95% CI, −0.04 to 0.19] percentage points).

### Sensitivity Analyses

Sensitivity analyses findings were generally consistent with the primary results (eResults, eTables 4-14 in Supplement 1). Using a logistic regression model, a 1% increase in childhood FFCR spending was associated with an 80% reduction in the odds (adjusted odds ratio, 0.20 [95% CI, 0.05-0.75]) of reporting adult fair or poor health (eTable 7 in Supplement 1). Differences between FFCR spending and overall health rating were greater in school districts within city boundaries than in independent school districts extending beyond city boundaries. Differences between FFCR spending and CVD were greater in respondents 36 to 59 years of age than in those 19 to 35 years of age.

## Discussion

In this retrospective cohort study of US urban residents, greater childhood exposure to FFCR spending was associated with a lower probability of reporting adult fair or poor health and CVD. These associations were primarily observed for public investments in primary and secondary education (for overall health and CVD), libraries (for overall health), and community development and housing (for CVD). These findings suggest that early-life public investments may yield long-term health benefits.

### Comparison to Prior Literature

Our findings align with ecological studies reporting that spending within the FFCR sectors correlates with improved area-level adult health, including better self-rated health and reduced CVD prevalence.^16,17,29^ However, area-level health data can obscure individual-level effects (ecological fallacy), particularly when resources or health benefits are unevenly distributed. Our use of individual-level outcomes mitigates these concerns.

We adopted a life-course perspective, which may yield more precise estimates of how investments shape health trajectories. A prior cross-sectional study found a 1% increase in county parks and recreation spending was associated with a 5% reduction in the odds of fair or poor health.^16^ Our sensitivity analyses suggest a 1% increase in childhood FFCR spending was associated with an 80% reduction in these odds in adulthood. Effect size differences may reflect variation in populations, baseline prevalence, covariate adjustment, or the spending measure. It may also suggest misspecification when not accounting for the long-term health associated with multiple early-life public investments. For example, schools are most relevant for young people and can influence indirect health-promoting pathways (e.g., health behaviors, socioeconomic mobility).^30,31^ Moreover, the public investment environment during adulthood may not reflect the one in childhood, particularly if they relocated after childhood. Even modest shifts in self-rated health are clinically meaningful: adults aged 40 to 54 years reporting fair or poor health face a 4- to 10-fold higher mortality hazard.^32^

Notably, our estimates were primarily associated with spending that was not on parks and recreation. Unlike past studies, we observed no associations between parks and recreation spending and the assessed health outcomes.^16,17,29^ In our sample, parks and recreation accounted for 3% of total expenditures, possibly limiting the power to detect long-term health effects. Spending levels alone may also not translate into equitable access to high-quality green spaces or recreational programming, potentially attenuating correlations.

We found no comparable studies for CVD effect sizes. Based on our findings, we can extrapolate estimates that suggest that a 1% increase (approximately $17 per capita) in childhood FFCR spending could prevent roughly 65 000 future CVD cases among children in the 95 cities studied (eDiscussion in Supplement 1 for calculations). The number needed to treat (12) and cost per case prevented ($7000) are comparable to smoking cessation interventions.^33,34^ This is notable given that heart disease remains the leading cause of death in the US, with annual costs exceeding $400 billion and projected to rise.^35,36^

We observed no association between FFCR spending and anxiety or depression diagnoses, contrasting with international evidence that has shown associations between other forms of social spending, such as employment and family benefits, and mental health outcomes.^37,38^ Underdiagnosis, underreporting, limited health care access, or lower US mental health funding could explain this difference in findings, but this explanation is less likely given our null sensitivity analysis with a psychological distress measure. Mental health is also shaped by genetic and interpersonal factors that may not be as responsive to FFCR spending.^39,40^

### Potential Mechanisms

Several pathways may explain associations between FFCR spending and health. School spending correlates with socioeconomic outcomes, which may impact health through health literacy, problem-solving skills, health behaviors, health care access, employment, and social networks.^26,41,42,43,44,45,46,47,48,49,50,51^ Library investments could similarly support positive academic and socioeconomic trajectories. School and library investments may foster relational health (eg, parent-child and teen programs), a favorable school climate, healthy habits, and access to quality health programming.^49^ The association between housing investments and CVD may reflect stable, affordable housing benefits, as housing instability is tied to adverse childhood experiences, mental and behavioral conditions, forgone medical care, and disrupted schooling.^52,53,54,55,56,57,58^ These mechanisms may operate independently or synergistically, and it is unclear if children benefit directly from these investments or through broader neighborhood effects.

### Implications

Policymakers could consider the long-term health spillover effects of public investments when making decisions during a funding surplus or deficit or altering the tax code. The Congressional Budget Office traditionally uses a 10-year window to evaluate a policy’s federal budgetary impact, but longer-term projections may be necessary for assessing childhood investments, including at the local level.^59^ The Congressional Budget Office found that the long-term fiscal effects of Medicaid for children could offset at least half of the initial costs.^59^ Our findings also highlight the collective impact of multiple child-centered investments.

Since education, parks, and libraries are primarily locally funded, state and federal redistributive policies may be needed to ensure equitable access to community resources and help offset lower local revenues or funding shortfalls.^1^ These efforts have the potential to promote health equity by addressing historical disinvestment in areas with higher racial segregation resulting from discriminatory policies and practices.^60,61^ For example, policies may be required to address public school funding disparities stemming from differences in property wealth across districts.^13^ States have attempted reforms for fairer education funding distribution across districts, which could be studied.^62,63^ Future research should investigate causal pathways, program-specific impacts, and effects in small and rural settings to inform targeted policy interventions.

### Limitations

Unmeasured confounding and selection bias are potential limitations in this study. We adjusted for childhood socioeconomic characteristics and residential moves to minimize these limitations. Findings may reflect broader city priorities or political or cultural beliefs rather than spending itself. Although we adjusted for the proportion of the city population living in poverty, public spending may also be a marker for other unequally distributed resources and concentrated wealth. FFCR spending may be correlated with public health and health care spending, although we adjusted for this spending. Centering on a different age could have produced different results, although ages 1 to 18 years were represented when data for 9 years of age were unavailable. We could not perform a dose-response analysis for the duration of exposure to spending levels. Data lacked program-level detail, and self-reported health outcomes could introduce bias. The focus on medium and large urban cities limits generalizability.

## Conclusions

Findings from this cohort study of US urban residents suggest that childhood FFCR public spending may have significant long-term influences on adult health. Investments in education, libraries, and community development and housing during childhood correlated with a lower likelihood of overall poor health or CVD in adulthood or both. These results underscore how local government spending may shape population health for the next generation.

## Notes

Supplement 1.
eMethods.
eResults.
eDiscussion.
**eTable 1.** Types of Expenditures by Functional Category **eTable 2.** Characteristics of Eligible Respondents in Weighted Study Sample, N = 2124 **eTable 3.** Unweighted and Unadjusted Regression Results for Multivariable Models of Health Outcomes on Fiscally Standardized City Operational Expenditures on Family-Focused Community Resources **eTable 4.** Survey Weighted and Adjusted Regression Results for Multivariable Models of Health Outcomes on Fiscally Standardized City Operational Expenditures on Family-Focused Community Resources: Sensitivity Analyses Using Alternative Independent Variable Constructions **eTable 5.** Survey Weighted and Adjusted Regression Results for Multivariable Models of Health Outcomes on Fiscally Standardized City Operational Expenditures on Family-Focused Community Resources: Sensitivity Analyses Using Alternative Dependent Variable Constructions **eTable 6.** Survey Weighted and Adjusted Regression Results for Multivariable Models of Health Outcomes on Fiscally Standardized City Operational Expenditures on Family-Focused Community Resources: Sensitivity Analyses Using Alternative Covariates **eTable 7.** Survey Weighted and Adjusted Regression Results for Multivariable Models of Health Outcomes on Fiscally Standardized City Operational Expenditures on Family-Focused Community Resources: Sensitivity Analysis Using an Alternative Logistic Regression Model **eTable 8.** Survey Weighted and Adjusted Regression Results for Multivariable Models of Health Outcomes on Fiscally Standardized City Operational Expenditures on Family-Focused Community Resources: Sensitivity Analysis Using an Alternative Sample Constructions **eTable 9.** Survey Weighted and Adjusted Regression Results for Multivariable Models of Health Outcomes on Fiscally Standardized City Operational Expenditures on Family-Focused Community Resources: Sensitivity Analysis Using Moderation by Childhood Residential Move **eTable 10.** Survey Weighted and Adjusted Regression Results for Multivariable Models of Health Outcomes on Fiscally Standardized City Operational Expenditures on Family-Focused Community Resources: Sensitivity Analysis Using Moderation by Age **eTable 11.** Survey Weighted and Adjusted Regression Results for Cardiovascular Outcome on Fiscally Standardized City Operational Expenditures on Family-Focused Community Resources: Stratified by Age **eTable 12.** Survey Weighted and Adjusted Regression Results for Multivariable Models of Health Outcomes on Fiscally Standardized City Operational Expenditures on Family-Focused Community Resources: Sensitivity Analysis Using Moderation by County Overlap and District Structure **eTable 13.** Survey Weighted and Adjusted Regression Results for Overall Health Rating Outcome on Fiscally Standardized City Operational Expenditures on Family-Focused Community Resources: Stratified by District Structure **eTable 14.** Survey Weighted and Adjusted Regression Results for Asthma Diagnosis on Fiscally Standardized City Operational Expenditures on Family-Focused Community Resources: Falsification Test

Supplement 2.
Data Sharing Statement

## References

[1] Minh A, Muhajarine N, Janus M, Brownell M, Guhn M. A review of neighborhood effects and early child development: how, where, and for whom, do neighborhoods matter? Health Place. 2017;46:155-174. doi:10.1016/j.healthplace.2017.04.012 28528276

[2] Shanahan KH, Subramanian SV, Burdick KJ, Monuteaux MC, Lee LK, Fleegler EW. Association of neighborhood conditions and resources for children with life expectancy at birth in the US. JAMA Netw Open. 2022;5(10):e2235912. doi:10.1001/jamanetworkopen.2022.35912 36239940 PMC9568807

[3] Aris IM, Perng W, Dabelea D, ; Program Collaborators for Environmental Influences on Child Health Outcomes. Associations of neighborhood opportunity and social vulnerability with trajectories of childhood body mass index and obesity among US children. JAMA Netw Open. 2022;5(12):e2247957. doi:10.1001/jamanetworkopen.2022.47957 36547983 PMC9857328

[4] Roubinov DS, Hagan MJ, Boyce WT, Adler NE, Bush NR. Family socioeconomic status, cortisol, and physical health in early childhood: the role of advantageous neighborhood characteristics. Psychosom Med. 2018;80(5):492-501. doi:10.1097/PSY.0000000000000585 29742755 PMC5976531

[5] Aris IM, Rifas-Shiman SL, Jimenez MP, . Neighborhood child opportunity index and adolescent cardiometabolic risk. Pediatrics. 2021;147(2):e2020018903. doi:10.1542/peds.2020-018903 33479165 PMC7906069

[6] Slopen N, Cosgrove C, Acevedo-Garcia D, Hatzenbuehler ML, Shonkoff JP, Noelke C. Neighborhood opportunity and mortality among children and adults in their households. Pediatrics. 2023;151(4):e2022058316. doi:10.1542/peds.2022-058316 36946099 PMC10624946

[7] Gennetian LA, Sanbonmatsu L, Katz LF, . The long-term effects of moving to opportunity on youth outcomes. Cityscape. 2012;14(2):137-167.

[8] Acevedo-Garcia D, McArdle N, Hardy EF, . The child opportunity index: improving collaboration between community development and public health. Health Aff (Millwood). 2014;33(11):1948-1957. doi:10.1377/hlthaff.2014.0679 25367989

[9] Acevedo-Garcia D, Lochner KA, Osypuk TL, Subramanian SV. Future directions in residential segregation and health research: a multilevel approach. Am J Public Health. 2003;93(2):215-221. doi:10.2105/AJPH.93.2.215 12554572 PMC1447719

[10] Williams DR, Collins C. Racial residential segregation: a fundamental cause of racial disparities in health. Public Health Rep. 2001;116(5):404-416. doi:10.1016/S0033-3549(04)50068-7 12042604 PMC1497358

[11] Acevedo-Garcia D, Osypuk TL, McArdle N, Williams DR. Toward a policy-relevant analysis of geographic and racial/ethnic disparities in child health. Health Aff (Millwood). 2008;27(2):321-333. doi:10.1377/hlthaff.27.2.321 18332486

[12] Allegretto S, García E, Weiss E. Public education funding in the US needs an overhaul: how a larger federal role would boost equity and shield children from disinvestment during downturns. Economic Policy Institute. 2022. Accessed December 1, 2024. https://www.epi.org/publication/public-education-funding-in-the-us-needs-an-overhaul/

[13] Brunner EJ, Schwegman D, Vincent JM. How much does public school facility funding depend on property wealth? Educ Finance Policy. 2023;18(1):25-51. doi:10.1162/edfp_a_00346

[14] McCullough JM, Leider JP. Government spending in health and nonhealth sectors associated with improvement in county health rankings. Health Aff (Millwood). 2016;35(11):2037-2043. doi:10.1377/hlthaff.2016.0708 27834244

[15] McCullough JM, Leider JP. The importance of health and social services spending to health outcomes in Texas, 2010-2016. South Med J. 2019;112(2):91-97. doi:10.14423/SMJ.0000000000000935 30708373 PMC6530967

[16] Tom Mueller J, Park SY, Mowen AJ. The relationship between self-rated health and local government spending on parks and recreation in the United States from 1997 to 2012. Prev Med Rep. 2018;13(13):105-112. 30568868 10.1016/j.pmedr.2018.11.018 PMC6297890

[17] Thorpe KE, Joski P. Association of social service spending, environmental quality, and health behaviors on health outcomes. Popul Health Manag. 2018;21(4):291-295. doi:10.1089/pop.2017.0136 29140747

[18] Melton-Fant C, Howard S, Cao X. Sex differences in the association between local government spending and mortality: evidence from Tennessee. South Med J. 2020;113(2):64-69. doi:10.14423/SMJ.0000000000001062 32016435

[19] Parker K, Horowitz JM, Brown A, Fry R. Cohn D’Vera, Igielnik R. Demographic and economic trends in urban, suburban and rural communities. Pew Research Center. May 22, 2018. Accessed December 18, 2024. https://www.pewresearch.org/social-trends/2018/05/22/demographic-and-economic-trends-in-urban-suburban-and-rural-communities/

[20] Strengthening the Reporting of Observational Studies in Epidemiology. STROBE. Accessed December 19, 2023, https://www.strobe-statement.org/

[21] Digest of education statistics, 2020. National Center for Education Statistics. Accessed April 6, 2025. https://nces.ed.gov/programs/digest/d20/tables/dt20_101.20.asp

[22] Panel Study of Income Dynamics. public use dataset and geocode match restricted use data. 2021. Accessed March 22, 2022. https://simba.isr.umich.edu/default.aspx

[23] Beaule A, Campbell F, Dascola M, . Institute for Social Research, Survey Research Center, Panel Study of Income Dynamics, University of Michigan. PSID main interview user manual: release 2021. Accessed July 26, 2022. https://psidonline.isr.umich.edu/data/Documentation/UserGuide2019.pdf

[24] National Historical Geographic Information System: about IPUMS NHGIS. Accessed December 18, 2024. https://www.nhgis.org/about-ipums-nhgis

[25] Manson S, Schroeder J, Van Riper D, Kugler T, Ruggles S. IPUMS National Historical Geographic Information System: Version 17.0 [dataset]. Minneapolis, MN: IPUMS. 2022. Accessed March 21, 2023. https://www.ipums.org/projects/ipums-nhgis/d050.V17.0

[26] MacKenzie T, Lebeaux R. Mortality versus municipal and state government spending in American cities. J Urban Health. 2021;98(5):665-675. doi:10.1007/s11524-021-00516-3 33761065 PMC8566648

[27] Lorem G, Cook S, Leon DA, Emaus N, Schirmer H. Self-reported health as a predictor of mortality: a cohort study of its relation to other health measurements and observation time. Sci Rep. 2020;10(1):4886. doi:10.1038/s41598-020-61603-0 32184429 PMC7078209

[28] Paradies Y, Ben J, Denson N, . Racism as a determinant of health: a systematic review and meta-analysis. PLoS One. 2015;10(9):e0138511. doi:10.1371/journal.pone.0138511 26398658 PMC4580597

[29] Mueller JT, Park SY, Mowen AJ. The relationship between parks and recreation per capita spending and mortality from 1980 to 2010: a fixed effects model. Prev Med Rep. 2019;14(100827). doi:10.1016/j.pmedr.2019.100827 30815338 PMC6377401

[30] Roshan S, Rahman F. Education and social mobility: a pathway to economic and social empowerment. Global Social Sciences Review. 2025;10(1):124-133. doi:10.31703/gssr.2025(X-I).11

[31] Daily SM, Mann MJ, Lilly CL, Bias TK, Smith ML, Kristjansson AL. School climate as a universal intervention to prevent substance use initiation in early adolescence: a longitudinal study. Health Educ Behav. 2020;47(3):402-411. doi:10.1177/1090198120914250 32281413 PMC7427833

[32] Dore EC, Idler E. Self-rated health predicts mortality–but it depends on your age. Soc Sci Med. 2024;362:117439. doi:10.1016/j.socscimed.2024.117439 39476729 PMC11585416

[33] Kahn R, Robertson RM, Smith R, Eddy D. The impact of prevention on reducing the burden of cardiovascular disease. Circulation. 2008;118(5):576-585. doi:10.1161/CIRCULATIONAHA.108.190186 18606915

[34] Barnett PG, Wong W, Hall S. The cost-effectiveness of a smoking cessation program for out-patients in treatment for depression. Addiction. 2008;103(5):834-840. doi:10.1111/j.1360-0443.2008.02167.x 18412763

[35] Kazi DS, Elkind MSV, Deutsch A, ; American Heart Association. Forecasting the economic burden of cardiovascular disease and stroke in the United States through 2050: a presidential advisory from the American Heart Association. Circulation. 2024;150(4):e89-e101. doi:10.1161/CIR.0000000000001258 38832515

[36] Heart disease facts. 2024. U.S. Centers for Disease Control and Prevention. Accessed November 29, 2024. https://www.cdc.gov/heart-disease/data-research/facts-stats/index.html

[37] Matsubayashi T, Sekijima K, Ueda M. Government spending, recession, and suicide: evidence from Japan. BMC Public Health. 2020;20(1):243. doi:10.1186/s12889-020-8264-1 32079525 PMC7033906

[38] Niedzwiedz CL, Mitchell RJ, Shortt NK, Pearce JR. Social protection spending and inequalities in depressive symptoms across Europe. Soc Psychiatry Psychiatr Epidemiol. 2016;51(7):1005-1014. doi:10.1007/s00127-016-1223-6 27138947 PMC4947487

[39] Melnychuk M, Zhylin M, Makarchuk N, Sakharova K, Shven Y. The influence of genetic markers on the development of mental disorders: a systematic review of studies. J Pioneer Med Sci. 2024;13(3):30-37. doi:10.61091/jpms202413306

[40] Pearce E, Birken M, Pais S, . Associations between constructs related to social relationships and mental health conditions and symptoms: an umbrella review. BMC Psychiatry. 2023;23(1):652. doi:10.1186/s12888-023-05069-0 37667255 PMC10478264

[41] Hamad R, Nguyen TT, Glymour MM, Vable A, Manly JJ, Rehkopf DH. Quality and quantity: the association of state-level educational policies with later life cardiovascular disease. Prev Med. 2019;126:105750. doi:10.1016/j.ypmed.2019.06.008 31195021 PMC6697595

[42] Baker BD. How money matters for schools. Learning Policy Institute. 2017. Accessed April 21, 2026. https://files.eric.ed.gov/fulltext/ED606469.pdf

[43] Baker B, Weber M. Beyond the echo-chamber: state investments and student outcomes in U.S. elementary and secondary education. Journal of Education Finance. 2016;42(1):1-27. doi:10.1353/jef.2016.a634272

[44] Greenwald R, Hedges LV, Laine RD. The effect of school resources on student achievement. Rev Educ Res. 1996;66(3):361-396. doi:10.3102/00346543066003361

[45] Han D, Kim S, Park H, Sohn H. Promoting student achievement in high school using school funding: evidence from quantile regression discontinuity design. Asia Pac Educ Rev. 2021;22:193-208. doi:10.1007/s12564-020-09667-5

[46] Sohn H, Park H, Jung H. The effect of extra school funding on students’ academic achievements under a centralized school financing system. Educ Finance Policy. 2023;18(1):1-24. doi:10.1162/edfp_a_00375

[47] Aryaman A, Jafar S, Mohamed A, . A cause and effect analysis: looking at the effects of lack of funding for schools on US students. Accessed April 29, 2026. 10.5281/zenodo.3983080

[48] Jackson CK, Johnson RC, Persico C. The effects of school spending on educational and economic outcomes: evidence from school finance reforms: NBER working paper No. 20847. National Bureau of Economic Research. 2015. Accessed December 1, 2024. https://www.nber.org/system/files/working_papers/w20847/w20847.pdf

[49] Suiter SV, Meadows ML. Educational attainment and educational contexts as social determinants of health. Prim Care. 2023;50(4):579-589. doi:10.1016/j.pop.2023.04.007 37866832

[50] Fletcher JM, Frisvold DE. Higher education and health investments: does more schooling affect preventive health care use? J Hum Cap. 2009;3(2):144-176. doi:10.1086/645090 22368727 PMC3285406

[51] Berkman ND, Sheridan SL, Donahue KE, Halpern DJ, Crotty K. Low health literacy and health outcomes: an updated systematic review. Ann Intern Med. 2011;155(2):97-107. doi:10.7326/0003-4819-155-2-201107190-00005 21768583

[52] Nobari TZ, Whaley SE. Severe housing-cost burden and low-income young children’s exposure to adverse experiences: a cross-sectional survey of WIC participants in Los Angeles County. Matern Child Health J. 2021;25(2):321-329. doi:10.1007/s10995-020-03032-z 33205312

[53] DeCandia CJ, Volk KT, Unick GJ. Evolving our understanding: housing instability as an ACE for young children. Advers Resil Sci. 2022;3(4):365-380. doi:10.1007/s42844-022-00080-y 36320362 PMC9607722

[54] Chandler CE, Austin AE, Shanahan ME. Association of housing stress with child maltreatment: a systematic review. Trauma Violence Abuse. 2022;23(2):639-659. doi:10.1177/1524838020939136 32677550 PMC7855012

[55] Marcal KE. The impact of housing instability on child maltreatment: a causal investigation. J Fam Soc Work. 2018;21(4-5):331-347. doi:10.1080/10522158.2018.1469563 30774282 PMC6377199

[56] Warren EJ, Font SA. Housing insecurity, maternal stress, and child maltreatment: an application of the family stress model. Soc Serv Rev. 2015;89(1):9-39. doi:10.1086/680043

[57] Hoyle ME, Chamberlain AW, Wallace D. The effect of home foreclosures on child maltreatment rates: a longitudinal examination of neighborhoods in Cleveland, Ohio. J Interpers Violence. 2022;37(5-6):NP2768-NP2790. doi:10.1177/0886260520943725 32723140

[58] Lal A, Slopen N. Housing instability and children’s health and education. JAMA Pediatr. 2024;178(10):967-968. doi:10.1001/jamapediatrics.2024.3172 39186283

[59] Ash E, Carrington W, Heller R, Hwang G. Exploring the effects of Medicaid during childhood on the economy and the budget: working paper 2023-07. November 2023. Congressional Budget Office. Accessed June 20, 2025. https://www.cbo.gov/system/files/2023-10/59231-Medicaid.pdf

[60] Blatt LR, Sadler RC, Jones EJ, Miller P, Hunter-Rue DS, Votruba-Drzal E. Historical structural racism in the built environment and contemporary children’s opportunities. Pediatrics. 2024;153(2):e2023063230. doi:10.1542/peds.2023-063230 38192230

[61] La Ferrara E, Mele A. Racial segregation and public school expenditure. Rochester, NY; 2006 Accessed January 27, 2025. https://papers.ssrn.com/abstract=926166

[62] Hoy S. How education is funded in California. Los Angeles Unified School District. Accessed May 1, 2022. https://www.lausd.org/cms/lib/CA01000043/Centricity/Domain/123/05_How%20Education%20is%20Funded%20in%20California.pdf

[63] Johnson R. School funding effectiveness: evidence from California’s local control funding formula. Learning Policy Institute; 2023 Aug:1-46. Report No. Accessed July 31, 2025. https://learningpolicyinstitute.org/product/school-funding-effectiveness-ca-lcff-report

