# Supplementary material for: Investments in Childhood Community Resources and Subsequent Adult Health Outcomes
Source: JAMA Netw Open. 2026 Jun 4;9(6):e2616711. doi: 10.1001/jamanetworkopen.2026.16711 (PMC13237617; doi:10.1001/jamanetworkopen.2026.16711)
Supplement: Supplement 1. — eMethods. eResults. eDiscussion. eTable 1. Types of Expenditures by Functional Category eTable 2. Characteristics of Eligible Respondents in Weighted Study Sample, N = 2124 eTable 3. Unweighted and Unadjusted Regression Results for Multivariable Models of Health Outcomes on Fiscally Standardized City Operational Expenditures on Family-Focused Community Resources eTable 4. Survey Weighted and Adjusted Regression Results for Multivariable Models of Health Outcomes on Fiscally Standardized City Operational Expenditures on Family-Focused Community Resources: Sensitivity Analyses Using Alternative Independent Variable Constructions eTable 5. Survey Weighted and Adjusted Regression Results for Multivariable Models of Health Outcomes on Fiscally Standardized City Operational Expenditures on Family-Focused Community Resources: Sensitivity Analyses Using Alternative Dependent Variable Constructions eTable 6. Survey Weighted and Adjusted Regression Results for Multivariable Models of Health Outcomes on Fiscally Standardized City Operational Expenditures on Family-Focused Community Resources: Sensitivity Analyses Using Alternative Covariates eTable 7. Survey Weighted and Adjusted Regression Results for Multivariable Models of Health Outcomes on Fiscally Standardized City Operational Expenditures on Family-Focused Community Resources: Sensitivity Analysis Using an Alternative Logistic Regression Model eTable 8. Survey Weighted and Adjusted Regression Results for Multivariable Models of Health Outcomes on Fiscally Standardized City Operational Expenditures on Family-Focused Community Resources: Sensitivity Analysis Using an Alternative Sample Constructions eTable 9. Survey Weighted and Adjusted Regression Results for Multivariable Models of Health Outcomes on Fiscally Standardized City Operational Expenditures on Family-Focused Community Resources: Sensitivity Analysis Using Moderation by Childhood Residential Move eTable 10. Survey Weighted and Adjusted Regression Results for M [file jamanetwopen-e2616711-s001.pdf]

Supplementary Online Content

La Charite J, Dudovitz R, Choi K, et al. Investments in childhood community resources and subsequent adult health outcomes. *JAMA Netw Open*. 2026;9(6):e2616711.
doi:10.1001/jamanetworkopen.2026.16711

eMethods.

eResults.

eDiscussion.

eTable 1. Types of Expenditures by Functional Category

eTable 2. Characteristics of Eligible Respondents in Weighted Study Sample, N = 2124

eTable 3. Unweighted and Unadjusted Regression Results for Multivariable Models of Health Outcomes on Fiscally Standardized City Operational Expenditures on Family-Focused Community Resources

eTable 4. Survey Weighted and Adjusted Regression Results for Multivariable Models of Health Outcomes on Fiscally Standardized City Operational Expenditures on Family-Focused Community Resources: Sensitivity Analyses Using Alternative Independent Variable Constructions

eTable 5. Survey Weighted and Adjusted Regression Results for Multivariable Models of Health Outcomes on Fiscally Standardized City Operational Expenditures on Family-Focused Community Resources: Sensitivity Analyses Using Alternative Dependent Variable Constructions

eTable 6. Survey Weighted and Adjusted Regression Results for Multivariable Models of Health Outcomes on Fiscally Standardized City Operational Expenditures on Family-Focused Community Resources: Sensitivity Analyses Using Alternative Covariates

eTable 7. Survey Weighted and Adjusted Regression Results for Multivariable Models of Health Outcomes on Fiscally Standardized City Operational Expenditures on Family-Focused Community Resources: Sensitivity Analysis Using an Alternative Logistic Regression Model

eTable 8. Survey Weighted and Adjusted Regression Results for Multivariable Models of Health Outcomes on Fiscally Standardized City Operational Expenditures on Family-Focused Community Resources: Sensitivity Analysis Using an Alternative Sample Constructions

© 2026 La Charite J et al. *JAMA Network Open*.

eTable 9. Survey Weighted and Adjusted Regression Results for Multivariable Models of Health Outcomes on Fiscally Standardized City Operational Expenditures on Family-Focused Community Resources: Sensitivity Analysis Using Moderation by Childhood Residential Move

eTable 10. Survey Weighted and Adjusted Regression Results for Multivariable Models of Health Outcomes on Fiscally Standardized City Operational Expenditures on Family-Focused Community Resources: Sensitivity Analysis Using Moderation by Age

eTable 11. Survey Weighted and Adjusted Regression Results for Cardiovascular Outcome on Fiscally Standardized City Operational Expenditures on Family-Focused Community Resources: Stratified by Age

eTable 12. Survey Weighted and Adjusted Regression Results for Multivariable Models of Health Outcomes on Fiscally Standardized City Operational Expenditures on Family-Focused Community Resources: Sensitivity Analysis Using Moderation by County Overlap and District Structure

eTable 13. Survey Weighted and Adjusted Regression Results for Overall Health Rating Outcome on Fiscally Standardized City Operational Expenditures on Family-Focused Community Resources: Stratified by District Structure

eTable 14. Survey Weighted and Adjusted Regression Results for Asthma Diagnosis on Fiscally Standardized City Operational Expenditures on Family-Focused Community Resources: Falsification Test

eReferences.

This supplementary material has been provided by the authors to give readers additional information about their work.

© 2026 La Charite J et al. *JAMA Network Open*.

eMethods.

Conceptual Frameworks

The Racialized Policy Feedback Framework and the Life Course Health Development Framework offer a conceptual basis for this work. The former posits that more decentralized policies—such as local government spending—allow for the variability that produces racial inequality and are thus more likely to funnel resources unevenly, contributing to differences in how benefits and burdens of policies are distributed, known as disproportionality.[1] This has been documented for public education, as funding primarily relies on state and local resources. On average, school districts in high-poverty areas have less per-pupil funding than districts in low-poverty areas.[2] The Life Course Health Development Framework helps conceptualize how these decentralized funding policies could have lifelong health implications. The framework proposes that one's health trajectory is shaped from childhood into adulthood when there is a mismatch between developmental demands and the environment, deterring normal healthy development and leading to disease.[3] For instance, if there are insufficient nearby community resources to meet a child's developmental needs (e.g., green space and opportunities for play), this may influence their health trajectory and contribute to disease. Combining these two frameworks provides a theoretical foundation for understanding how differences in exposure to local public spending during childhood can influence differential health outcomes over time.

Setting

The inclusion criterion that participants needed to have lived in a city with at least 150,000 residents in 1980 before age 18 was selected because it is one of the inclusion criteria in the Fiscally Standardized City (FiSC) public finance database. Out of 103 eligible cities, 95 (92%) were included in our sample. The cities that were not included were those where a Panel Study of Income Dynamics (PSID) 2019 core survey respondent did not live during the specified years.

Data Sources and Dataset Construction

© 2026 La Charite J et al. *JAMA Network Open*.

FiSC Database (local public expenditures). The FiSC database combines data from municipal, county, special district, and school district governments to create FiSC city-level revenue and expenditure estimates.[2] This allows for comparisons of local government finances since local governments differ in how they allocate revenues and expenditures among multiple local governments. To calculate the FiSC estimates, the city government revenues and expenditures are combined with the proportion of revenues and expenditures of its overlying counties, school districts, and special districts allocated to cover the population of total residents or students within the central city (e.g. if a city accounts for 20% of the county population, than 20% of revenues and expenditures for the county will be allocated to the FiSC).[2,3] The database uses a standardized classification system and collection processes for local government finances, which have been relatively stable since the 1970s, and converts estimates to current dollars.[4]

The FiSC dataset includes two samples of U.S. cities-- the core FiSC sample and the legacy FiSC sample. The core FiSC samples include 150 cities based on the following criteria: the two largest cities in each state and all cities with populations of 150,000 or more in 1980 and 200,000 or more in 2010.[4] The Legacy FiSC sample includes 95 cities with population declines of at least 20 percent from their peak, poverty rates exceeding the national average, and a peak population of at least 50,000. In total, there are 212 cities across the two city samples.[4] A list of all the cities, including those under each criterion, is published elsewhere.[5–7]

PSID Panel. The PSID panel sample initially consisted of 5,000 households and has since expanded to over 9,500 families and 24,000 individuals.[8] Through a telephone-based core survey conducted every two years, it collects demographic and household characteristics, economic well-being measures, and health information. PSID staff interview one person per family and collect information on each family member, with greater detail obtained for the reference person (adults with primary financial responsibility for the family unit) and their married/cohabiting partner, if any.[8]

PSID 2019 Core Survey (adult health outcomes). We drew health-outcome data from the PSID 2019 core survey, which included 9,569 families and 26,084 individuals, yielding a response rate of 90%.[8] However, not all 26,084 individuals were eligible for this study. To be eligible, the individuals had

to be a reference person or their spouse/partner for whom the health outcome measures were collected (n=14,827). Among these individuals, only the PSID panel sample members would have information on their childhood (n=9,698). A subsample of these individuals would be born between 1960 and 2000. Moreover, none of the 2017-2019 new immigrant sample would be eligible for selection, as they joined the PSID study sample in 2017 and would not have childhood information. This left an eligible sample of 2,204 adults prior to merging with the public finance dataset.

We selected the 2019 PSID core survey since it was the most recent wave available when data merging for this study began, corresponded with the most recent FiSC public finance data, and predated potential confounding from the COVID-19 pandemic.

PSID Geospatial Data (data source linkage by geography). The PSID has geospatial data, including Federal Information Processing System (FIPS) place codes for its panel members. FIPS place codes are numbers that uniquely identify a U.S. incorporated place independent of any county (e.g., an independent city, parish, borough, or township). We used the FIPS place codes and the year when the PSID respondent was age nine (or the closest age to nine if data for their age nine were unavailable) to link the PSID records to the FiSC and IPUMS-NHGIS.

IPUMS-NHGIS (city-level covariates). We identified city-level demographic data from the IPUMS-NHGIS, a data hub for national census data that provides summary tables and time-series data on population, housing, agriculture, and economics at all levels of U.S. census geography.[9]

Participants

The complex sample design, recruitment, and follow-up for the PSID are described elsewhere.[5] The oldest eligible participant could be age 17 in 1977 (FiSC data starting year), and the youngest eligible participant could be age 19 in 2019 (the most recent childhood linkage year could be age 17 from the 2017 PSID primary survey). This left 2,204 adult PSID respondents.

The subsequent filter identified adults whose place code during their childhood in the PSID matched a place code in the FiSC database (n=2164). Forty respondents were dropped because they lived in Hawaii or New Jersey, which have state-administered schools.

© 2026 La Charite J et al. *JAMA Network Open*.

Finally, we narrowed to adults with complete data for the public spending exposure variable, primary outcome variable (overall health rating), and covariates (n=2124) to allow model comparisons in the model-building process. See Figure 2 for the sample flow diagram.

Variables

Exposure Variable: Public Spending (childhood). The exposure variable was defined as the sum of city and proportional county, school, and special district operational spending per capita on family-focused community resource (FFCR) spending in childhood (continuous variable). See eTable 1 for what each FFCR spending category includes. Our primary and secondary public education spending variable includes state grant funding channeled through the school district. We selected these spending categories to focus on non-healthcare-associated spending and because programs and services delivered through these sectors are particularly relevant to families and children and are hypothesized to influence childhood experiences. For the primary analysis, we focused on the magnitude of expenditures to better capture differences across cities, particularly those stemming from lower revenues in poorer municipalities.

We focused on public spending when the individual was approximately nine years old. If we did not have data for the individual when they were 9 years old, we selected the childhood age closest to 9 with a matching FIPS place code to maximize sample size. In the few cases in which we did not have data at age nine but had data for two ages equidistant from it (e.g., ages 8 and 10), we randomly selected one of the two available ages. We could not combine spending across childhood (birth to age 17) because PSID respondents had varying numbers of recorded geocodes during that period.

Adult Health Outcome Variables (young to middle adulthood). We selected cardiovascular disease as the physical health outcome since it is the leading cause of death.[10] We similarly chose depression and anxiety as the mental health outcomes, as they are the most common causes of mental illness among adults.[11] These adult health outcomes have also been associated with both adverse and positive childhood experiences.[12–15]

For cardiovascular disease, the PSID asked respondents whether a doctor or other health professional ever diagnosed them with a (1) stroke, (2) heart attack, (3) coronary heart disease, angina,

© 2026 La Charite J et al. *JAMA Network Open*.

congestive heart failure, or (4) hypertension. We combined responses to items 1-4 into a single dichotomous variable indicating whether the respondent reported being diagnosed with any cardiovascular condition.

For the mental health outcomes, the PSID asked respondents whether a doctor or other health professional ever diagnosed the participant with any emotional, nervous, or psychiatric condition. If reported, the PSID then asked about the specific diagnosis. We created an indicator variable for whether the respondent endorsed ever being diagnosed with depression or anxiety.

Covariates: City-level (childhood). We created variables for city features in the linkage year to control for city demographics that may influence the degree of city spending on FFCR, as well as contextual factors that may impact health. City-level covariate measures were derived from decennial Census time-series data in IPUMS-NHGIS. We used the decennial values since we required data for years predating the more frequent American Community Survey (ACS). We used the decennial year closest to the data linkage year. For linkage years 2015 and 2017, we used the ACS since the 2020 Census decennial data were not complete at the city/place code level at the time of analysis. Population size was defined as small (< 100,000 residents), medium (100,000-250,000 residents), or large (> 250,000 residents). Based on the distributions of the variables, we transformed population density, median rent, and percent foreign born to the natural log scale, and dichotomized the percent of the city population with a high school diploma at the median. To isolate spending on FFCRs, we controlled for other spending by summing FiSC operational spending across the remaining sectors after removing FFCR spending for the linkage year (see eTable 1 for what was included). We divided this variable into quartiles due to concerns about multicollinearity with the exposure variable.

Covariates: Individual level (childhood). We adjusted for the individual's childhood socioeconomic status, as it could influence where parents choose to raise their children and the individual's health trajectory. In PSID primary survey records spanning 1977-2017, respondents' parents reported their household income when the respondents were children. We developed a six-level childhood household income variable for the linkage year (less than 100% of the federal poverty limit (FPL), 100-

© 2026 La Charite J et al. *JAMA Network Open*.

199% FPL, 200-299% FPL, 300-399% FPL, 400% or more FPL, unknown) that is relative to the household size and the linkage year's FPL. In the 2019 PSID primary survey, PSID respondents were asked how much education their mother and father completed and whether they moved from birth to age 16. We created a dichotomous variable indicating whether the individual had moved before age 17. We created a seven-level categorical variable (out-of-country education, less than high school, high school graduate or GED, some college or vocational school, college graduate, some or completed graduate school, unknown) for the highest educational attainment of the mother and father.

Covariates: Individual level (adulthood). We constructed individual-level sociodemographic variables relevant to the health outcomes examined. Individual-level covariates were obtained from the 2019 PSID primary survey. We included sex (male, female) and adult marital status (married vs. never married vs. widowed, divorced, annulled, or separated), whether a family member in their adult household had health insurance (yes/no). Race/ethnicity (Hispanic/Latinx, Non-Hispanic Asian/Pacific Islander, Non-Hispanic Black, Non-Hispanic White, Non-Hispanic Other) was self- or proxy-reported by a household family member and included to adjust for ways racism can influence childhood environments and health.[19] The 'Other' race category included Non-Hispanic American Indian, Non-Hispanic Alaskan Native, Non-Hispanic Multi-Racial, or, if the respondent selected "Other" as their race. We also included an indicator for whether the covariates and outcomes were self-reported or proxy-reported by another household family member.

Statistical Methods

Bivariate Analyses. We conducted bivariate Pearson's chi-squared and Two-sample t-test analyses to compare respondents living in childhood cities with FFCR spending below and above the median (eTable 2).

Multivariable Primary Analyses. Linear probability models apply ordinary least squares regression to model the probability of a binary outcome; coefficients are interpreted as absolute percentage point differences. We selected this regression model for ease of interpretation and performance for the overall health rating measure.[20,21] These models adjusted for childhood and adult demographic

© 2026 La Charite J et al. *JAMA Network Open*.

variables, childhood city-level characteristics, and operational spending on other sectors, apart from the FFCRs, to account for variables we hypothesized could be related to childhood city of residence and local spending, as well as adult health. We considered a multilevel modeling approach or clustered standard errors to account for observations within the same city, but ultimately decided against it because some cities had only one or a few observations, and observations from the same city were often from different years. Instead, we adjusted for multiple relevant city-level features.

Missing and Follow-Up. The public finance information in the FiSC database was complete. Prior to publishing the FiSC database, the FiSC authors attempted to minimize measurement errors with data review, linear interpolations or missing data imputations.[4] Of note, public finance information from Hawaii and New Jersey was excluded from the analysis because they have state-sponsored schools without local school district variation and were thus not eligible. For the IPUMS-Census data, a subsample of cities lacked median gross rent data for 1980. To fill in this missing data, we used single imputation based on the median gross rent for the city in 1990 and 2000 and our city-level covariates to estimate the median gross rent in 1980. For the PSID data, based on the initial study inclusion criteria, 8% of respondents were missing data for one of the covariates or the primary outcome before we created the "unknown" category for missing information for the individual childhood variables (See Figure 1).

Sensitivity Analyses. *Alternative Exposure Variables* (*see eTable 4*) - We evaluated the following alternative exposure variable constructions: (i) we rescaled FFCR spending to $100 per capita, (ii) we used a continuous proportion of total operational spending allocated to FFCRs, and (iii) we used a continuous percentile of FFCR spending relative to the rest of the cities in the public finance database for the linkage year to account for secular trends in public spending. We also tested standardized individual spending category variables (z-scores) because expenditure magnitudes varied across categories.

Alternative Dependent Variables (see eTable 5) - We transformed the overall health rating variable for the outcome variables into discrete values on a continuous scale (Poor = 0.40, Fair = 0.71, Good = 0.84, Very Good = 0.93, Excellent = 0.98) based on prior literature.[21] Since most of the individuals with the CVD outcome were composed of respondents with hypertension, we also tested

hypertension as the outcome on its own. As an alternative to the anxiety or depression diagnosis outcome, we also tested psychological distress as an outcome based on symptoms reported on six items of the Kessler-6 psychological distress scale during the 2019 PSID survey. We dichotomized at the published threshold of a score of 13, which is predictive of a clinically diagnosable mental health condition.

Covariates (see eTable 6) - We also maintained operational spending on other sectors, minus FFCR spending, as a continuous variable on a natural log scale rather than dividing it into quantiles. Since race could also serve as a proxy for residential segregation, we ran the models without the race/ethnicity covariate.

Regression Modeling (see eTable 7) - We also tested an alternative modeling approach using logistic regression.

Sample Construction (see eTable 8)- As a sensitivity analysis of the sample construction, we excluded participants for whom the survey was completed by proxy to ensure the accuracy of responses (sample size dropped to 1972). Since a new wave of PSID data was released before manuscript submission, we repeated the primary analysis using a sample constructed from the 2021 PSID Core Survey Wave. We replaced the 2019-reported health outcomes with the 2021-reported health outcomes.

Moderation by Age, Residential Move, Government Structure, and District Structure - We tested for moderation by childhood residential move, age in years (continuous), government structure (whether the city has an overlying county government), and district structure (dependent school district, independent school district within city bounds, independent school district extending beyond city bounds). If the interaction term was significant, we stratified the results.

We examined whether the associations differed in strength between respondents who reported a childhood residential move and those who did not (see eTable 9). We could not differentiate whether these residential moves occurred within or across cities. We wanted to assess whether it was appropriate to combine individuals who reported moving and those who did not in the same sample for the primary analysis, since we were only including public spending data from a single point in time during childhood.

© 2026 La Charite J et al. *JAMA Network Open*.

We tested whether the associations between FFCR spending and the health outcomes were moderated by age. The age analysis was conducted to explore the potential effect of secular trends and to test our hypothesis that the association between childhood exposure to FFCR public spending and physical health may be stronger among older respondents (eTable 10 & 11).

We also examined whether our findings were robust to differences in local governance structures. Some cities operate under an overlying county government, while others do not, which may influence our measure of FFRC spending by varying spending patterns across cities and counties. In addition, the strength of the associations may differ depending on how a school district is governed and how the district's boundaries relate to city boundary lines. School districts could be controlled by a city government (city-dependent) or by a county government (county-dependent). The school district could also operate independently of a city or county government, with its own elected school board, but exist within the city or county boundary lines (city-wide or county-wide independent) or extend beyond city lines (an independent district whose boundaries extend beyond city boundaries). To account for these possibilities, we constructed two governance type variables. First, we created a binary indicator denoting whether a city is situated within an overlying county government. Second, we developed a three-category measure of school district type. The public finance database identifies five school district types: (1) city-dependent, (2) county-dependent, (3) city-wide independent, (4) county-wide independent, and (5) independent districts whose boundaries extend beyond city boundaries. Given small cell sizes for the county-dependent and county-independent school district types, we combined the county-dependent with the city-dependent and the county-independent with the city-independent types, resulting in three analytic categories. We then conducted two moderation analyses. First, we interacted the binary county overlay variable with the natural log of per capita FFCR spending for each of the three outcomes. Second, we interacted the three-level school district type variable with the natural log of per capita FFCR spending for each of the three outcomes.

Falsification Test (eTable 14) – To validate our results, we retested the model with an alternative outcome we expected to yield a null result. We selected asthma diagnosis as a negative outcome. Asthma

© 2026 La Charite J et al. *JAMA Network Open*.

diagnosis is not a perfect negative control, as it is influenced by environment and lifestyle, but it has a high genetic contribution of 40-70%,[24] and diagnosis is less influenced by environmental factors than measuring asthma control.

© 2026 La Charite J et al. *JAMA Network Open*.

eResults.

Sensitivity Analyses

Sensitivity analysis findings were consistent with the primary results, except that the association between the proportion of total operational spending allocated to FFCRs and CVD became null (p=0.91).

The association between FFCR spending and CVD was stronger among respondents aged 36-59 years (−0.36 percentage point difference, 95% CI: −0.65 to −0.07) compared to respondents aged 19-35 years (−0.15 percentage point difference, 95% CI: −0.34 to 0.03). The association between FFCR spending and overall health rating was stronger in school districts within city boundaries (-0.28 percentage point difference, 95% CI: -0.63 to -0.06 for city/county dependent districts; -0.28 percentage point difference, 95% CI: -0.53 to -0.03 for city/county wide independent districts) than in independent school districts extending beyond city boundaries (-0.11 percentage point difference, 95% CI: -40 to 0.18). This finding strengthens our primary results, as the jurisdictions with the least measurement error are those with the strongest identified associations. The strength of the associations was consistent whether the city had an overlying county government or not. Using asthma diagnosis as a negative control produced the expected null result.

© 2026 La Charite J et al. *JAMA Network Open*.

eDiscussion.

Calculations

If we multiply the average population under age 18 years (1,044,767 average population x 26% below age 18 = 271,639) for the 95 cities in the sample (25,805,745 child residents) by the regression percentage point difference (0.25%), our findings would translate to an estimated decrease of about 64,514 city residents becoming diagnosed with cardiovascular disease before age 60 for a 1% increase in FFCR spending in childhood (~$17 per capita). The average spending on community resources for the study sample was $1,725, ranging from $835 to $3,948. Therefore, if all 95 cities spent an additional ~$17 per capita above their average spending on community resources, it could reduce the number of children later diagnosed with cardiovascular disease by nearly 65,000.

We found an absolute risk reduction of about 8.5% in reporting CVD for individuals exposed to public spending on community resources at the 75th percentile compared to the 25th percentile. This translates to a number needed to treat (NNT) of 12. As a point of comparison, the 30-year NNT is 13 for smoking cessation on myocardial infarction.[25] The absolute dollar difference between spending at the 25th and 75th percentiles was $584 per capita. Therefore, the cost to prevent 1 CVD case would be about $7,000 in public dollars (NNT 12 x $584) allocated to community resources for every 12 individuals to prevent one case of CVD. In comparison, the average cost for smoking cessation services was $346 or estimated to be $5,170 per life-year.[26]

© 2026 La Charite J et al. *JAMA Network Open*.

eTable 1. Types of Expenditures by Functional Category

**Elementary and secondary education** (Census function code 12)	**Housing & Community Development** (Census function code 50)	**Libraries** (Census function code 52)	**Parks and Recreation** (Census function code 61)	**Other Sector Spending Minus the Family-Focused Community Resource Sectors**
Administration & supervision of the public-school system including qualified charter schools, special education, Head start, vocational education, school-based libraries, employees involved in school lunch, student activities, community services, pupil transit, health services, guidance, counseling, maintenance, and operation.<br><br>For the purposes of this study, we only included operational spending from this category. Non-operational (i.e., capital) spending from this category was not included.	Planning, construction, furnishing, & operation of public housing projects, rent subsidies, housing & mortgage finance agencies, promotion of home ownership, assistance for repair & renovation of existing homes, programs to encourage private sector housing production. Urban renewal and slum clearance; redevelopment & rehabilitation of substandard or deteriorated facilities & areas; rural redevelopment; revitalization of commercial areas.<br><br>For the purposes of this study, we only included operational spending from this category. Non-operational (i.e., capital) spending from this category was not included.	Establishment and provision of libraries for use by the general public, programs to promote, development and coordinate library services and facilities.<br><br>For the purposes of this study, we only included operational spending from this category. Non-operational (i.e., capital) spending from this category was not included.	Playgrounds, courts/courses for recreational sports, public beaches, swimming pools, camping areas, galleries/museums, zoos, gardens, auditoriums, stadiums, recreational and convention centers, community reactional and cultural activities and programming that operate outside the school system.<br><br>For the purposes of this study, we only included operational spending from this category. Non-operational (i.e., capital) spending from this category was not included.	Includes operational spending on the following sectors: Other welfare, higher education, hospitals, public health, transportation, public safety, natural resources, sewage, solid waste management, governmental administration, interest on general debt, utilities, liquor store, employee retirement trust expenditures after removing the operational spending on community-based support resources.<br><br>For the purposes of this study, we only included operational spending from this category. Non-operational (i.e., capital) spending from this category was not included.

Caption: N/a
Source: Federal, state, and local governments: government finance and employment classification manual. U.S. Census Bureau. April 24, 2001. Accessed February 2, 2022. https://www2.census.gov/govs/class/classfull.pdf

© 2026 La Charite J et al. *JAMA Network Open*.

eTable 2. Characteristics of Eligible Respondents in Weighted Study Sample, N = 2124

	Total	Below median for public spending^	Above median for public spending^	
	N= 2124	N= 1073 (51%)	N= 1051 (49%)	
	(N) weighted %	(N) weighted %	(N) weighted %	*P* value
**Childhood Individual-Level Variables**				
**Parent highest educational attainment**				
Outside the country	(67) 6%	(11) 2%	(56) 10%	<.001
Less than high school	(215) 7%	(120) 7%	(95) 8%	
High school graduate	(706) 27%	(354) 29%	(352) 26%	
Some college or vocational	(529) 23%	(249) 21%	(280) 25%	
College graduate	(359) 21%	(175) 20%	(184) 21%	
Graduate school	(191) 14%	(120) 19%	(71) 9%	
Unknown	(57) 2%	(44) 2%	(13) 2%	
**Childhood household income**				
<100% Federal Poverty Level	(654) 20%	(302) 16%	(352) 24%	<.001
100-199% Federal Poverty Level	(524) 22%	(255) 18%	(269) 27%	
200-299% Federal Poverty Level	(360) 18%	(197) 20%	(163) 15%	
300-399% Federal Poverty Level	(239) 17%	(134) 18%	(105) 15%	
400% or more Federal Poverty Level	(280) 22%	(159) 26%	(121) 16%	
Unknown	(67) 2%	(26) 2%	(41) 3%	
**Move during childhood**				
Did not move during childhood	(1,708) 79%	(891) 82%	(817) 76%	0.03
Moved during childhood	(416) 21%	(182) 18%	(234) 24%	
**Adulthood Individual-Level Variables**				
Age in Years in 2019 (m, SD) [range]	38.9 ± 10.2 [19-59 years]	43.5 ± 8.5	33.1 ± 9.1	<.001
**Sex**				
Female	(1,223) 52%	(447) 47%	(454) 49%	0.61
Male	(901) 48%	(626) 53%	(597) 51%	
**Race/Ethnicity**				
Asian or Pacific Islander	(21) 2%	(6) 1%	(15) 3%	<.001
Black	(1,335) 33%	(674) 31%	(661) 35%	
Hispanic/Latinx	(177) 14%	(49) 9%	(128) 21%	
White	(483) 47%	(305) 55%	(178) 36%	
Other or Multiracial	(108) 4%	(39) 4%	(69) 5%	
**Educational attainment**				
Less than high school	(221) 7%	(111) 7%	(110) 7%	0.22
High school graduate or GED	(674) 28%	(314) 26%	(360) 29%	
Some college or vocational	(683) 30%	(349) 29%	(334) 32%	
College graduate	(294) 18%	(151) 19%	(143) 18%	
Graduate school	(252) 17%	(148) 19%	(104) 14%	
**Marital status**				
Married	(677) 43%	(408) 51%	(269) 33%	<.001
Never married	(1,096) 42%	(414) 30%	(682) 58%	
Widowed, divorced, annulled, separated	(351) 14%	(251) 19%	(100) 33%	
**Adulthood household income**				
<100% Federal Poverty Level	(398) 13%	(185) 10%	(213) 15%	<.01
100-199% Federal Poverty Level	(436) 17%	(173) 12%	(263) 22%	
200-299% Federal Poverty Level	(339) 14%	(185) 14%	(154) 13%	
300-399% Federal Poverty Level	(292) 14%	(155) 15%	(137) 12%	
400% or more Federal Poverty Level	(659) 43%	(375) 48%	(284) 38%	
**Family health insurance**				

Yes	(1,863) 91%	(938) 92%	(925) 90%	0.38
**Respondent: Self or By Proxy**				
Self	(1,976) 89%	(968) 85%	(1008) 94%	<.001
**Overall health rating**				
Fair or poor health	(389) 17%	(228) 19%	(161) 15%	0.14
**Cardiovascular disease diagnosis**				
Yes	(458) 22%	(320) 28%	(138) 13%	<.001
**Anxiety or depression diagnosis**				
Yes	(184) 10%	(96) 10%	(88) 10%	0.77
**City Demographics**				
**City Population Size**				
Midsize (population 100,000-250,000)	(470) 24%	(299) 25%	(171) 23%	0.61
Large (population >250,000)	(1,654) 76%	(774) 75%	(880) 77%	
**Percent of City Population**				
Less than 18 years old (m, SD)	0.26 ± 0.03	0.26 ± 0.03	0.26 ± 0.04	0.08
At least a high school diploma/GED[%] (m, SD)	0.48 ± 0.21	0.47 ± 0.20	0.49 ± 0.20	0.13
Below the Federal Poverty Level (m, SD)	0.17 ± 0.05	0.15 ± 0.04	0.20 ± 0.06	<.001
Unemployed (m, SD)	0.08 ± 0.03	0.07 ± 0.03	0.09 ± 0.03	<.001
Non-Hispanic White (m, SD)	0.56 ± 0.20	0.63 ± 0.16	0.47 ± 0.20	<.001
Foreign born (m, SD)	0.12 ± 0.12	0.10 ± 0.08	0.16 ± 0.14	<.001
**Population density**[#] (m, SD)	0.02 ± 0.04	0.03 ± 0.04	0.02 ± 0.04	<.001
**Median gross rent** (m, SD)	897 ± 194	876 ± 180	924 ± 210	<.001
**Standardized City Spending Variables**				
Total spending (m, SD)	4,063 ± 1,345	3,388 ± 867	4,936 ± 1,394	<.001
Family-focused community resource (FFCR) spending (m, SD)	1,725 ± 428	1,429 ± 191	2,106 ± 352	<.001
Proportion of total spending on FFCRs (m, SD)	0.44 ± 0.07	0.44 ± 0.07	0.44 ± 0.08	0.05
Education spending (m, SD)	1,424 ± 364	1,191 ± 165	1,725 ± 343	<.001
Library spending (m, SD)	36.70 ± 16.5	33 ± 14	41 ± 18	<.001
Parks & recreation spending (m, SD)	114 ± 60	101 ± 48	132 ± 71	<.001
Community development & housing spending (m, SD)	150 ± 116	105 ± 116	208 ± 140	<.001

Caption: m = mean; SD = standard deviation; GED = General Educational Development
FFCR = family-focused community resources = public primary and secondary education, libraries, parks and recreation, and community development and housing
[%] Percent of city population that is 25 years and older with at least a high school diploma/GED
[#] Population density is the average population per square mile
All dollar amounts are per capita and adjusted to be comparable to 2019 dollars
^ Above/Below median for public spending – defined as above and below median for the operational spending on family-focused community resources (public primary and secondary education, libraries, parks and recreation, and community development and housing)
Source: Authors' analysis of data from the Panel Study of Income Dynamics 1977-2019 Core Surveys, Fiscally Standardized Cities Database 1977-2017, Integrated Public Use Microdata Series National Historical Geographic Information System 1980-2017.

© 2026 La Charite J et al. *JAMA Network Open*.

eTable 3. Unweighted and Unadjusted Regression Results for Multivariable Models of Health Outcomes on Fiscally Standardized City Operational Expenditures on Family-Focused Community Resources

	**Outcome 1** Fair or Poor Health		**Outcome 2** Cardiovascular Disease Diagnosis		**Outcome 3** Anxiety or Depression Diagnosis	
	n=2124		n=2124		n=2120	
	PPD (95% CI)[a.b]	*P* value	PPD (95% CI)[a.b]	*P* value	PPD (95% CI)[a.b]	*P* value
**Changes to survey weight and covariate adjustment**						
Unweighted, Unadjusted	−0.10 (−0.17 to −0.04)	0.001	−0.37 (−0.44 to −0.31)	<.000	-0.01 (−0.06 to 0.03)	0.56
Unweighted, Adjusted	−0.08 (−0.19 to 0.03)	0.13	−0.13 (-0.24 to −0.01)	0.03	0.00 (−0.08 to 0.09)	0.91
Weighted[c], Unadjusted	−0.12 (−0.21 to −0.03)	0.01	−0.38 (−0.49 to −0.26)	<.000	−0.03 (−0.11 to 0.04)	0.41

Caption: PPD = percentage point difference; CI = confidence interval

[a] We used linear probability regression models. The displayed values are interpreted as the percentage point change in the outcome for a 1% increase in FFCR spending.

[b] All shown analyses adjusted for covariates: parent highest parental educational attainment, childhood household income, childhood move, childhood city population size, city demographics [% less than 18 years, poverty, unemployed, non-Hispanic White, foreign born], population density, median rent adjusted to be comparable to 2019 dollars, age in 2019, sex, race/ethnicity, adult educational attainment, marital status, adult household income, family health insurance, whether self or proxy respondent, public operational spending on the remaining sectors divided into quartiles.

[c] Model 3 used the Panel Study of Income Dynamics 2019 longitudinal survey weight.

Source: Authors' analysis of data from the Panel Study of Income Dynamics 1977-2019 Core Surveys, Fiscally Standardized Cities Database 1977-2017, Integrated Public Use Microdata Series National Historical Geographic Information System 1980-2017.

© 2026 La Charite J et al. *JAMA Network Open*.

eTable 4. Survey Weighted and Adjusted Regression Results for Multivariable Models of Health Outcomes on Fiscally Standardized City Operational Expenditures on Family-Focused Community Resources: **Sensitivity Analyses Using Alternative Independent Variable Constructions**

	**Outcome 1**		**Outcome 2**		**Outcome 3**	
	**Fair or Poor Health**		**Cardiovascular Disease Diagnosis**		**Anxiety or Depression Diagnosis**	
	n=2124		n=2124		n=2120	
	PPD (95% CI)[a.b]	*P* value	PPD (95% CI)[a.b]	*P* value	PPD (95% CI)[a.b]	*P* value
**Alternative independent variables**						
Family-focused community resource spending, $100 per capita	−0.01 (-0.02 to −0.004)	0.003	−0.01 (−0.02 to −0.00)	0.02	0.00 (−0.00 to 0.01)	0.37
Proportion of total operational spending on family-focused community resources	−0.50 (−0.94 to −0.05)	0.03	−0.03 (−0.52 to 0.47)	0.91	0.25 (−0.07 to 0.57)	0.12
Percentile of a city's family-focused community resource spending per capita relative to other cities in that particular year	−0.13 (−0.25 to 0.00)	0.05	−0.16 (−0.29 to 0.03)	0.02	0.08 (−0.01 to 0.18)	0.10
Standardized public education spending	−0.04 (−0.07, −0.00)	0.03	−0.06 (−0.10, −0.02)	0.006	0.02 (−0.01, 0.05)	0.22
Standardized library spending	−0.04 (−0.07, −0.00)	0.03	0.00 (−0.03, 0.03)	0.94	0.00 (−0.02, 0.02)	0.97
Standardized parks and recreation spending	−0.03 (−0.05, 0.00)	0.10	0.00 (−0.03, 0.03)	0.80	0.00 (−0.02, 0.03)	0.80
Standardized community development and housing spending	0.02 (−0.01, 0.04)	0.22	−0.05 (−0.08, −0.01)	0.009	0.01 (−0.02, 0.03)	0.65

Caption: PPD = percentage point difference; CI = confidence interval

[a] We used linear probability regression models. We applied the Panel Study of Income Dynamics 2019 longitudinal survey weights. The displayed values are interpreted as the percentage point change in the outcome for a 1% increase in FFCR spending.

[b] All shown analyses adjusted for covariates: parent highest parental educational attainment, childhood household income, childhood move, childhood city population size, city demographics [% less than 18 years, poverty, unemployed, non-Hispanic White, foreign born], population density, median rent adjusted to be comparable to 2019 dollars, age in 2019, sex, race/ethnicity, adult educational attainment, marital status, adult household income, family health insurance, whether self or proxy respondent, public operational spending on the remaining sectors divided into quartiles.

Source: Authors' analysis of data from the Panel Study of Income Dynamics 1977-2019 Core Surveys, Fiscally Standardized Cities Database 1977-2017, Integrated Public Use Microdata Series National Historical Geographic Information System 1980-2017.

© 2026 La Charite J et al. *JAMA Network Open*.

eTable 5. Survey Weighted and Adjusted Regression Results for Multivariable Models of Health Outcomes on Fiscally Standardized City Operational Expenditures on Family-Focused Community Resources: **Sensitivity Analyses Using Alternative Dependent Variable Constructions**

**Outcome 1**		**Outcome 2**		**Outcome 3**	
Overall health rating, transformed to discrete values on continuous scale (Poor = 0.40, Fair = 0.71, Good = 0.84, Very Good = 0.93, Excellent = 0.98)		Hypertension only		Psychological Distress Meeting clinical threshold for clinically meaningfully psychological distress on Kessler-6 Scale	
n=2124		n=2124		n=2120	
PPD (95% CI)[a.b]	*P* value	PPD (95% CI) [a.b]	*P* value	PPD (95% CI) [a.b]	*P* value
0.06 (0.01 to 0.11)	0.02	−0.24 (−0.42 to −0.06)	0.009	-0.05 (−0.12 to 0.02)	0.20

Caption: PPD = percentage point difference; CI = confidence interval

[a] We used linear probability regression models. We applied the Panel Study of Income Dynamics 2019 longitudinal survey weights. The values are interpreted as the percentage point change in the outcome for a 1% increase in FFCR spending.

[b] All shown analyses adjusted for covariates: parent highest parental educational attainment, childhood household income, childhood move, childhood city population size, city demographics [% less than 18 years, poverty, unemployed, non-Hispanic White, foreign born], population density, median rent adjusted to be comparable to 2019 dollars, age in 2019, sex, race/ethnicity, adult educational attainment, marital status, adult household income, family health insurance, whether self or proxy respondent, public operational spending on the remaining sectors divided into quartiles.

Source: Authors' analysis of data from the Panel Study of Income Dynamics 1977-2019 Core Surveys, Fiscally Standardized Cities Database 1977-2017, Integrated Public Use Microdata Series National Historical Geographic Information System 1980-2017.

eTable 6. Survey Weighted and Adjusted Regression Results for Multivariable Models of Health Outcomes on Fiscally Standardized City Operational Expenditures on Family-Focused Community Resources: **Sensitivity Analyses Using Alternative Covariates**

	**Outcome 1**		**Outcome 2**		**Outcome 3**	
	**Fair or Poor Health**		**Cardiovascular Disease Diagnosis**		**Anxiety or Depression Diagnosis**	
	n=2124		n=2124		n=2120	
	PPD (95% CI)[a.b]	*P* value	PPD (95% CI)[a.b]	*P* value	PPD (95% CI)[a.b]	*P* value
**Alternative covariates**						
Operational spending on other sectors as continuous log variable	−0.25 (−0.42 to −0.08)	0.004	−0.22 (−0.42 to 0.02)	0.03	0.08 (−0.05 to 0.21)	0.21
Without percent of city non-Hispanic White	−0.26 (−0.43 to −0.09)	0.002	−0.22 (−0.42 to 0.02)	0.03	0.09 (−0.04 to 0.22)	0.19

Caption: PPD = percentage point change; CI = confidence interval

[a] We used linear probability regression models. We applied the Panel Study of Income Dynamics 2019 longitudinal survey weights. The displayed values are interpreted as the percentage point change in the outcome for a 1% increase in FFCR spending.

[b] All shown analyses adjusted for covariates: parent highest parental educational attainment, childhood household income, childhood move, childhood city population size, city demographics [% less than 18 years, poverty, unemployed, non-Hispanic White, foreign born], population density, median rent adjusted to be comparable to 2019 dollars, age in 2019, sex, adult educational attainment, marital status, adult household income, family health insurance, whether self or proxy respondent, public operational spending on continuous scale.

Source: Authors' analysis of data from the Panel Study of Income Dynamics 1977-2019 Core Surveys, Fiscally Standardized Cities Database 1977-2017, Integrated Public Use Microdata Series National Historical Geographic Information System 1980-2017.

eTable 7. Survey Weighted and Adjusted Regression Results for Multivariable Models of Health Outcomes on Fiscally Standardized City Operational Expenditures on Family-Focused Community Resources: **Sensitivity Analysis Using an Alternative Logistic Regression Model**

	**Outcome 1**		**Outcome 2**		**Outcome 3**	
	**Fair or Poor Health**		**Cardiovascular Disease Diagnosis**		**Anxiety or Depression Diagnosis**	
	n=2124		n=2124		n=2120	
	aOR (95% CI)[a.b]	*P* value	aOR (95% CI)[a.b]	*P* value	aOR (95% CI)[a.b]	*P* value
**Logistic regression model and predicted margins**						
Logistic Regression	0.20 (0.05 to 0.75)	0.02	0.13 (0.03 to 0.53)	0.004	2.16 (0.50 to 9.40)	0.31
Margins dy/dx)/ Predicted Probability[c]	−0.20 (−0.36 to −0.03)		−0.28 (−0.47 to −0.09)		0.06 (−0.06 to 0.18)	

Caption: aOR = adjusted odds ratio; CI = confidence interval

[a] We used logistic regression models. We applied the Panel Study of Income Dynamics 2019 longitudinal survey weights.

[b] All shown analyses adjusted for covariates: parent highest parental educational attainment, childhood household income, childhood move, childhood city population size, city demographics [% less than 18 years, poverty, unemployed, non-Hispanic White, foreign born], population density, median rent adjusted to be comparable to 2019 dollars, age in 2019, sex, race/ethnicity, adult educational attainment, marital status, adult household income, family health insurance, whether self or proxy respondent, public operational spending on the remaining sectors divided into quartiles.

[c] Margins estimated from ± 0.05 mean of the natural log of operational spending on family-focused community resources

Source: Authors' analysis of data from the Panel Study of Income Dynamics 1977-2019 Core Surveys, Fiscally Standardized Cities Database 1977-2017, Integrated Public Use Microdata Series National Historical Geographic Information System 1980-2017.

© 2026 La Charite J et al. *JAMA Network Open*.

eTable 8. Survey Weighted and Adjusted Regression Results for Multivariable Models of Health Outcomes on Fiscally Standardized City Operational Expenditures on Family-Focused Community Resources: **Sensitivity Analysis Using an Alternative Sample Constructions**

	**Outcome 1**		**Outcome 2**		**Outcome 3**	
	**Fair or Poor Health**		**Cardiovascular Disease Diagnosis**		**Anxiety or Depression Diagnosis**	
	PPD (95% CI)[a.b]	*P* value	PPD (95% CI)[a.b]	*P* value	PPD (95% CI)[a.b]	*P* value
**Alternative Sample Constructions**						
Sample narrowed to respondents who self-reported their responses (rather than proxy report from their spouse/partner), n=1972	−0.22 (−0.38 to −0.06)	0.006	−0.22 (−0.41 to -0.03)	0.02	0.02 (−0.10 to 0.14)	0.77
2021 Panel Study of Income Dynamics Core Survey Sample, n=1835	−0.16 (−0.30 to −0.02)	0.03	−0.29 (−0.47 to −0.11)	0.002	−0.02 (−0.16 to 0.11)	0.74

Caption: PPD = percentage point difference; CI = confidence interval

[a] We used linear probability regression models. We applied the Panel Study of Income Dynamics 2019 longitudinal survey weights. The displayed values are interpreted as the percentage point change in the outcome for a 1% increase in FFCR spending.

[b] All shown analyses adjusted for covariates: parent highest parental educational attainment, childhood household income, childhood move, childhood city population size, city demographics [% less than 18 years, poverty, unemployed, non-Hispanic White, foreign born], population density, median rent adjusted to be comparable to 2019 dollars, age in 2019, sex, race/ethnicity, adult educational attainment, marital status, adult household income, family health insurance, whether self or proxy respondent, public operational spending on the remaining sectors divided into quartiles.

[c] We applied the Panel Study of Income Dynamics 2031 longitudinal survey weights.

Source: Authors' analysis of data from the Panel Study of Income Dynamics 1977-2019 Core Surveys, Fiscally Standardized Cities Database 1977-2017, Integrated Public Use Microdata Series National Historical Geographic Information System 1980-2017.

© 2026 La Charite J et al. *JAMA Network Open*.

eTable 9. Survey Weighted and Adjusted Regression Results for Multivariable Models of Health Outcomes on Fiscally Standardized City Operational Expenditures on Family-Focused Community Resources: **Sensitivity Analysis Using Moderation by Childhood Residential Move**

	**Outcome 1**		**Outcome 2**		**Outcome 3**	
	**Fair or Poor Health**		**Cardiovascular Disease Diagnosis**		**Anxiety or Depression Diagnosis**	
	n=2124		n=2124		n=2120	
	PPD (95% CI)[a.b]	*P* value	PPD (95% CI)[a.b]	*P* value	PPD (95% CI)[a.b]	*P* value
**Moderation by any childhood residential move**						
Operational spending on family-focused community resources, ln scale	−0.21 (−0.38 to −0.05)	0.01	−0.28 (−0.47 to −0.09)	0.005	0.04 (−0.09 to 0.17)	0.57
Any childhood residential move	−0.59 (−2.16 to 0.98)	0.46	−0.71 (−2.75 to 1.34)	0.50	−1.04 (−2.17 to 0.08)	0.70
Interaction term: Spending & any moves	0.09 (−0.12 to 0.30)	0.42	0.11 (−0.17 to 0.38)	0.44	0.14 (−0.01 to 0.30)	0.06
Wald Test F	F = 0.65	0.42	F = 0.59	0.44	F = 3.45	0.06

Caption: PPD = percentage point difference; CI = confidence interval.
[a] We used linear probability regression models. We applied the Panel Study of Income Dynamics 2019 longitudinal survey weights. The displayed values are interpreted as the percentage point change in the outcome for a 1% increase in FFCR spending.
[b] All shown analyses adjusted for covariates: parent highest parental educational attainment, childhood household income, childhood city population size, city demographics [% less than 18 years, poverty, unemployed, non-Hispanic White, foreign born], population density, median rent adjusted to be comparable to 2019 dollars, age in 2019, sex, race/ethnicity, adult educational attainment, marital status, adult household income, family health insurance, whether self or proxy respondent, public operational spending on the remaining sectors divided into quartiles.
[c] We applied the Panel Study of Income Dynamics 2031 longitudinal survey weights.
Source: Authors' analysis of data from the Panel Study of Income Dynamics 1977-2019 Core Surveys, Fiscally Standardized Cities Database 1977-2017, Integrated Public Use Microdata Series National Historical Geographic Information System 1980-2017.

© 2026 La Charite J et al. *JAMA Network Open*.

eTable 10. Survey Weighted and Adjusted Regression Results for Multivariable Models of Health Outcomes on Fiscally Standardized City Operational Expenditures on Family-Focused Community Resources: **Sensitivity Analysis Using Moderation by Age**

	**Outcome 1**		**Outcome 2**		**Outcome 3**	
	**Fair or Poor Health**		**Cardiovascular Disease Diagnosis**		**Anxiety or Depression Diagnosis**	
	n=2124		n=2124		n=2120	
	PPD (95% CI)[a.b]	*P* value	PPD (95% CI)[a.b]	*P* value	PPD (95% CI)[a.b]	*P* value
**Moderation by Age**						
Operational spending on public education, ln scale	−0.31 (−0.70 to 0.08)	0.12	0.23 (−0.19 to 0.65)	0.28	0.01 (−0.03 to 0.35)	0.94
Age in years, continuous	−0.02 (−0.07 to 0.03)	0.62	0.10 (0.02 to 0.19)	0.02	−0.01 (−0.08 to 0.05)	0.75
Interaction term between spending & age	0.00 (−0.01 to 0.01)	0.54	−0.01 (−0.02 to −0.00)	0.03	0.00 (−0.01 to 0.01)	0.74
Wald Test F	F = 0.39	0.53	F = 4.62	0.03	F = 0.11	0.73

Caption: PPD = percentage point difference; CI = confidence interval

[a] We used linear probability regression models. We applied the Panel Study of Income Dynamics 2019 longitudinal survey weights. The displayed values are interpreted as the percentage point change in the outcome for a 1% increase in FFCR spending.

[b] All shown analyses adjusted for covariates: parent highest parental educational attainment, childhood household income, childhood move, childhood city population size, city demographics [% less than 18 years, poverty, unemployed, non-Hispanic White, foreign born], population density, median rent adjusted to be comparable to 2019 dollars, sex, race/ethnicity, adult educational attainment, marital status, adult household income, family health insurance, whether self or proxy respondent, public operational spending on the remaining sectors divided into quartiles.

Source: Authors' analysis of data from the Panel Study of Income Dynamics 1977-2019 Core Surveys, Fiscally Standardized Cities Database 1977-2017, Integrated Public Use Microdata Series National Historical Geographic Information System 1980-2017.

© 2026 La Charite J et al. *JAMA Network Open*.

eTable 11. Survey Weighted and Adjusted Regression Results for Cardiovascular Outcome on Fiscally Standardized City Operational Expenditures on Family-Focused Community Resources: **Stratified by Age**

	**Outcome 2**	
	**Cardiovascular Disease Diagnosis**	
	n=2124	
	PPD (95% CI) [a,b]	*P* value
Age 30 or less in 2019	−0.15 (−0.34, 0.03)	0.11
Age over 30 years in 2019	−0.36 (−0.65 to −0.07)	0.02

Caption: PPD = percentage point difference; CI = confidence interval

[a] We used linear probability regression models. We applied the Panel Study of Income Dynamics 2019 longitudinal survey weights. The displayed values are interpreted as the percentage point change in the outcome for a 1% increase in FFCR spending.

[b] All shown analyses adjusted for covariates: parent highest parental educational attainment, childhood household income, childhood move, childhood city population size, city demographics [% less than 18 years, poverty, unemployed, non-Hispanic White, foreign born], population density, median rent adjusted to be comparable to 2019 dollars, sex, race/ethnicity, adult educational attainment, marital status, adult household income, family health insurance, whether self or proxy respondent, public operational spending on the remaining sectors divided into quartiles.

Source: Authors' analysis of data from the Panel Study of Income Dynamics 1977-2019 Core Surveys, Fiscally Standardized Cities Database 1977-2017, Integrated Public Use Microdata Series National Historical Geographic Information System 1980-2017.

© 2026 La Charite J et al. *JAMA Network Open*.

eTable 12. Survey Weighted and Adjusted Regression Results for Multivariable Models of Health Outcomes on Fiscally Standardized City Operational Expenditures on Family-Focused Community Resources: **Sensitivity Analysis Using Moderation by County Overlap and District Structure**

	**Outcome 1**		**Outcome 2**		**Outcome 3**	
	**Fair or Poor Health**		**Cardiovascular Disease Diagnosis**		**Anxiety or Depression Diagnosis**	
	n=2124		n=2124		n=2120	
	PPD (95% CI)[a.b]	*P* value	PPD (95% CI)[a.b]	*P* value	PPD (95% CI)[a.b]	*P* value
**Moderation by county overlap**						
Operational spending on family-focused community resources, ln scale	−0.31 (−0.51 to −0.12)	0.002	−0.15 (−0.38 to −0.08)	0.20	0.04 (−0.10 to 0.18)	0.57
County overlay	−1.02 (−2.53 to 0.50)	0.19	1.32 (−0.54 to 3.19)	0.17	−0.23 (−1.39 to 0.92)	0.69
Interaction term: Spending & county overlay	0.13 (−0.07 to 0.34)	0.19	−0.18 (−0.43 to 0.07)	0.16	0.04 (−0.12 to 0.19)	0.63
Wald Test F	F = 1.72	0.19	F = 1.96	0.16	F = 0.23	0.63
**Moderation by district structure**						
Operational spending on family-focused community resources, ln scale	−0.48 (−0.71 to −0.26)	<.001	−0.11 (−0.36 to 0.14)	0.39	0.05 (−0.12 to 0.21)	0.56
District structure: City or county dependent	Reference		Reference		Reference	
City or county wide independent	-2.59 (−4.54 to -0.63)	0.01	0.93 (−1.33 to 3.18)	0.42	−0.41 (−1.80 to 0.98)	0.57
Independent schools	-2.21 (−4.32 to -0.25)	0.03	1.67 (−0.47 to 3.80)	0.13	−0.26 (−1.59 to 1.07)	0.70
Interaction term: Spending & City or county dependent	Reference		Reference		Reference	
Interaction term: Spending & City or county wide independent	0.34 (0.08 to 0.60)	0.01	−0.12 (−0.42 to 0.18)	0.44	0.06 (−0.13 to 0.24)	0.54
Interaction term: Spending & Independent schools	0.29 (0.03, 0.55)	0.03	−0.22 (−0.51 to 0.06)	0.12	0.04 (−0.14 to 0.22)	0.65
Wald Test F	3.63	0.03	1.21	0.30	0.21	0.81

Caption: PPD = percentage point difference; CI = confidence interval

[a] We used linear probability regression models. We applied the Panel Study of Income Dynamics 2019 longitudinal survey weights. The values are interpreted as the percentage point change in the outcome for a 1% increase in FFCR spending.

© 2026 La Charite J et al. *JAMA Network Open*.

[b] All shown analyses adjusted for covariates: parent highest parental educational attainment, childhood household income, childhood move, childhood city population size, city demographics [% less than 18 years, poverty, unemployed, non-Hispanic White, foreign born], population density, median rent adjusted to be comparable to 2019 dollars, age in 2019, sex, race/ethnicity, adult educational attainment, marital status, adult household income, family health insurance, whether self or proxy respondent, public operational spending on the remaining sectors divided into quartiles.
Source: Authors' analysis of data from the Panel Study of Income Dynamics 1977-2019 Core Surveys, Fiscally Standardized Cities Database 1977-2017, Integrated Public Use Microdata Series National Historical Geographic Information System 1980-2017.

eTable 13. Survey Weighted and Adjusted Regression Results for Overall Health Rating Outcome on Fiscally Standardized City Operational Expenditures on Family-Focused Community Resources: **Stratified by District Structure**

	**Outcome 1**	
	**Overall Health Rating**	
	n=2124	
	PPD (95% CI) [a.b]	*P* value
City or county dependent district	−0.28 (−0.63 to 0.06)	0.11
City or county wide independent	−0.28 (−0.53 to −0.03)	0.03
Independent schools beyond boundaries	−0.11 (−0.40 to 0.18)	0.44

Caption: PPD = percentage point difference; CI = confidence interval
[a] We used linear probability regression models. We applied the Panel Study of Income Dynamics 2019 longitudinal survey weights. The displayed values are interpreted as the percentage point change in the outcome for a 1% increase in FFCR spending.
[b] All shown analyses adjusted for covariates: parent highest parental educational attainment, childhood household income, childhood move, childhood city population size, city demographics [% less than 18 years, poverty, unemployed, non-Hispanic White, foreign born], population density, median rent adjusted to be comparable to 2019 dollars, age in 2019, sex, race/ethnicity, adult educational attainment, marital status, adult household income, family health insurance, whether self or proxy respondent, public operational spending on the remaining sectors divided into quartiles.
Source: Authors' analysis of data from the Panel Study of Income Dynamics 1977-2019 Core Surveys, Fiscally Standardized Cities Database 1977-2017, Integrated Public Use Microdata Series National Historical Geographic Information System 1980-2017.

eTable 14. Survey Weighted and Adjusted Regression Results for **Asthma Diagnosis** on Fiscally Standardized City Operational Expenditures on Family-Focused Community Resources: **Falsification Test**

	**Outcome**	
	**Asthma Diagnosis**	
	n=2122	
	PPD (95% CI)[a.b]	*P* value
Operational spending on family-focused community resources, ln scale	0.02 (−0.12 to 0.17)	0.74

Caption: PPD = percentage point difference; CI = confidence interval
[a] We used linear probability regression models. We applied the Panel Study of Income Dynamics 2019 longitudinal survey weights. The displayed values are interpreted as the percentage point change in the outcome for a 1% increase in FFCR spending.
[b] All shown analyses adjusted for covariates: parent highest parental educational attainment, childhood household income, childhood move, childhood city population size, city demographics [% less than 18 years, poverty, unemployed, non-Hispanic White, foreign born], population density, median rent adjusted to be comparable to 2019 dollars, age in 2019, sex, race/ethnicity, adult educational attainment, marital status, adult household income, family health insurance, whether self or proxy respondent, public operational spending on the remaining sectors divided into quartiles.
Source: Authors' analysis of data from the Panel Study of Income Dynamics 1977-2019 Core Surveys, Fiscally Standardized Cities Database 1977-2017, Integrated Public Use Microdata Series National Historical Geographic Information System 1980-2017.

eReferences.

1. Michener J. Policy feedback in a racialized polity. Policy Stud J. 2019 Mar 19;47(2):423–50. doi:https://doi.org/10.1111/psj.12328

2. Allegretto S, García E, Weiss E. Public education funding in the US needs an overhaul: How a larger federal role would boost equity and shield children from disinvestment during downturns. Econ Policy Inst. 2022 Jul 12;47.

3. Halfon N, Hochstein M. Life course health development: An integrated framework for developing health, policy, and research. Milbank Q. 2002;80(3):433–79. doi:10.1111/MILQ.2002.80.ISSUE-3

4. Langley AH. Lincoln Institute of Land Policy [Internet]. 2020 [cited 2022 Jul 18]. Methodology used to create fiscally standardized cities database working paper WP20AL1. Available from: https://www.lincolninst.edu/publications/working-papers/methodology-used-create-fiscally-standardized-cities-database-0/

5. Langley A. Lincoln Institute of Land Policy [Internet]. 2020 [cited 2022 Jul 18]. 212 cities in Fiscally Standardized Cities Database. Available from: https://www.lincolninst.edu/app/uploads/legacy-files/gwipp/upload/files/FiSC/list_of_fiscs.pdf

6. Langley A. Lincoln Institute of Land Policy [Internet]. 2020 [cited 2022 Jul 18]. 95 cities in legacy cities sample of the Fiscally Standardized Cities Database. Available from: https://www.lincolninst.edu/app/uploads/legacy-files/gwipp/upload/files/FiSC/list_of_fiscs_legacy_cities_sample.pdf

7. Langley A. Lincoln Institute of Land Policy [Internet]. 2022 [cited 2022 Jul 18]. 150 cities in core FiSC sample of the Fiscally Standardized Cities Database. Available from: https://www.lincolninst.edu/app/uploads/legacy-files/gwipp/upload/files/FiSC/list_of_fiscs_core_fisc_sample.pdf

8. Beaule A, Campbell F, Dascola M, Insolera N, Johnson D, Juska P, et al. Institute for Social Research, University of Michigan [Internet]. 2021 [cited 2022 Jul 26]. PSID main interview user manual: Release 2021. Available from: https://psidonline.isr.umich.edu/data/Documentation/UserGuide2019.pdf PubMed PMID: 25246403.

9. University of Minnesota. IPUMS NHGIS | National Historical Geographic Information System [Internet]. 2026 [cited 2024 Dec 18]. About IPUMS NHGIS. Available from: https://www.nhgis.org/about-ipums-nhgis

10. FastStats - leading causes of death [Internet]. [cited 2025 Jan 22]. Available from: https://www.cdc.gov/nchs/fastats/leading-causes-of-death.htm

11. NAMI: National Alliance on Mental Illness [Internet]. [cited 2022 Mar 25]. Mental Health By the Numbers | NAMI: National Alliance on Mental Illness. Available from: https://www.nami.org/mhstats

12. Allen H, Wright BJ, Vartanian K, Dulacki K, Li HF. Examining the Prevalence of Adverse Childhood Experiences and Associated Cardiovascular Disease Risk Factors Among Low-Income Uninsured

Adults. Circ Cardiovasc Qual Outcomes. 2019 Sep;12(9):e004391. doi:10.1161/CIRCOUTCOMES.117.004391 PubMed PMID: 31450964.

13. Bellis MA, Hughes K, Ford K, Ramos Rodriguez G, Sethi D, Passmore J. Life course health consequences and associated annual costs of adverse childhood experiences across Europe and North America: a systematic review and meta-analysis. Lancet Public Health. 2019 Oct 1;4(10):e517–28. doi:10.1016/S2468-2667(19)30145-8 PubMed PMID: 31492648.

14. Slopen N, Chen Y, Guida JL, Albert MA, Williams DR. Positive childhood experiences and ideal cardiovascular health in midlife: Associations and mediators. Prev Med. 2017 Apr 1;97:72–9. doi:10.1016/J.YPMED.2017.01.002 PubMed PMID: 28087467.

15. Bethell C, Jones J, Gombojav N, Linkenbach J, Sege R. Positive Childhood Experiences and Adult Mental and Relational Health in a Statewide Sample: Associations Across Adverse Childhood Experiences Levels. JAMA Pediatr. 2019 Nov 4;173(11):e193007. doi:10.1001/jamapediatrics.2019.3007

16. Bethell C, Jones J, Gombojav N, Linkenbach J, Sege R. Positive childhood experiences and adult mental and relational health in a statewide sample: Associations across adverse childhood experiences levels. JAMA Pediatr. 2019 Nov 1;173(11). doi:10.1001/jamapediatrics.2019.3007 PubMed PMID: 31498386.

17. Su S, Jimenez MP, Roberts CTF, Loucks EB. The Role of Adverse Childhood Experiences in Cardiovascular Disease Risk: a Review with Emphasis on Plausible Mechanisms. Curr Cardiol Rep. 2015 Aug 20;17(10):88. doi:10.1007/s11886-015-0645-1

18. Sahle BW, Reavley NJ, Li W, Morgan AJ, Yap MBH, Reupert A, et al. The association between adverse childhood experiences and common mental disorders and suicidality: an umbrella review of systematic reviews and meta-analyses. Eur Child Adolesc Psychiatry. 2022 Oct 1;31(10):1489–99. doi:10.1007/s00787-021-01745-2

19. Paradies Y, Ben J, Denson N, Elias A, Priest N, Pieterse A, et al. Racism as a Determinant of Health: A Systematic Review and Meta-Analysis. Hills RK, editor. PLOS ONE. 2015 Sep 23;10(9):e0138511. doi:10.1371/journal.pone.0138511

20. Lumley T, Diehr P, Emerson S, Chen L. The importance of the normality assumption in large public health data sets. Annu Rev Public Health. 2002 May;23(1):151–69. doi:10.1146/annurev.publhealth.23.100901.140546

21. Doorslaer EV, Jones AM. Inequalities in self-reported health: validation of a new approach to measurement. J Health Econ. 2003 Jan;22(1):61–87. doi:10.1016/S0167-6296(02)00080-2

22. Kessler RC, Barker PR, Colpe LJ, Epstein JF, Gfroerer JC, Hiripi E, et al. Screening for Serious Mental Illness in the General Population. Arch Gen Psychiatry. 2003;60(2):184–9. doi:10.1001/archpsyc.60.2.184

23. Kessler RC, Andrews G, Colpe LJ, Hiripi E, Mroczek DK, Normand SLT, et al. Short screening scales to monitor population prevalences and trends in non-specific psychological distress. Psychol Med. 2002 Aug;32(6):959–76. doi:10.1017/S0033291702006074 PubMed PMID: 12214795.

© 2026 La Charite J et al. *JAMA Network Open*.

24. Koppelman GH, Pino-Yanes M, Melén E, Powell P, Bracke KR, Celedón JC, et al. Genetic and environmental risk factors for asthma: towards prevention. Lancet Respir Med. 2025 Nov 1;13(11):1011–25. doi:10.1016/S2213-2600(25)00256-5

25. Kahn R, Robertson RM, Smith R, Eddy D. The impact of prevention on reducing the burden of cardiovascular disease. Circulation. 2008 Jul 7;118(5):576–85. doi:https://doi.org/10.1161/CIRCULATIONAHA.108.190186 open_in_new

26. Barnett PG, Wong W, Hall S. The cost-effectiveness of a smoking cessation program for out-patients in treatment for depression. Addict Abingdon Engl. 2008 May;103(5):834–40. doi:10.1111/j.1360-0443.2008.02167.x PubMed PMID: 18412763.

© 2026 La Charite J et al. *JAMA Network Open*.
