# Supplementary material for: Investments in Childhood Community Resources and Subsequent Adult Health Outcomes
Source: JAMA Netw Open. 2026 Jun 4;9(6):e2616711. doi: 10.1001/jamanetworkopen.2026.16711 (PMC13237617; doi:10.1001/jamanetworkopen.2026.16711)
Supplement: Supplement 2. — Data Sharing Statement [file jamanetwopen-e2616711-s002.pdf]

Data Sharing Statement

La Charite. Investments in Childhood Community Resources and Subsequent Adult Health Outcomes. *JAMA Netw Open*. Published June 04, 2026.
doi:10.1001/jamanetworkopen.2026.16711

Data
Data available: No

Additional Information
Explanation for why data not available: The data for this analysis used a combination of publicly available and restricted data files. Publicly available data files can be downloaded from the websites for the Panel Study of Income Dynamics, the IPUMS National Historical Geographic Information System, and the Lincoln Institute of Land Policy Fiscally Standardized Cities. The restricted Panel Study of Income Dynamics data may be accessed through a Virtual Data Enclave through an application process.
